# Supplementary material for: ‘Optimising PharmacoTherapy In the multimorbid elderly in primary CAre’ (OPTICA) to improve medication appropriateness: study protocol of a cluster randomised controlled trial
Source: BMJ Open. 2019 Sep 3;9(9):e031080. doi: 10.1136/bmjopen-2019-031080 (PMC6731954; doi:10.1136/bmjopen-2019-031080)
Supplement: Author's manuscript [file bmjopen-2019-031080.draft_revisions.pdf]

BMJ Open is committed to open peer review. As part of this commitment we make the peer review history of every article we publish publicly available.

When an article is published we post the peer reviewers' comments and the authors' responses online. We also post the versions of the paper that were used during peer review. These are the versions that the peer review comments apply to.

The versions of the paper that follow are the versions that were submitted during the peer review process. They are not the versions of record or the final published versions. They should not be cited or distributed as the published version of this manuscript.

BMJ Open is an open access journal and the full, final, typeset and author-corrected version of record of the manuscript is available on our site with no access controls, subscription charges or pay-per-view fees (http://bmjopen.bmj.com).

If you have any questions on BMJ Open's open peer review process please email
info.bmjopen@bmj.com

BMJ Open

Design of 'Optimizing PharmacoTherapy In the Multimorbid Elderly in Primary Care' (OPTICA) to improve medication appropriateness: a cluster randomised controlled trial

Journal:	*BMJ Open*
Manuscript ID	bmjopen-2019-031080
Article Type:	Protocol
Date Submitted by the Author:	15-Apr-2019

Complete List of Authors:	Jungo, Katharina; Institute of Primary Health Care (BIHAM), University of Bern,<br>Rozsnyai, Zsofia; Institute of Primary Health Care (BIHAM), University of Bern<br>Mantelli, Sophie; Institute of Primary Health Care (BIHAM), University of Bern<br>Floriani, Carmen; Institute of Primary Health Care (BIHAM), University of Bern<br>Löwe, Axel; Institute of Primary Health Care (BIHAM), University of Bern; Department of General Internal Medicine, Inselspital, Bern University Hospital, University of Bern<br>Lindemann, Fanny; Institute of Primary Health Care (BIHAM), University of Bern<br>Schwab, Nathalie; Institute of Primary Health Care (BIHAM), University of Bern; Department of General Internal Medicine, Inselspital, Bern University Hospital, University of Bern<br>Meier, Rahel; Universität Zürich, Institute of Primary Care, University of Zurich<br>Elloumi, Lamia; Department of Information and Computing Sciences, Utrecht University; Department of Communication and Cognition, Tilburg University<br>Huibers, Corlina; Department of Geriatric Medicine, University Medical Centre Utrecht<br>Sallevelt, Bastiaan; Department of Clinical Pharmacy, University Medical Centre Utrecht<br>Meulendijk, Michiel; Department of Public Health and Primary Care, Leiden University Medical Center, Leiden<br>Reeve, Emily; Quality Use of Medicines and Pharmacy Research Centre, School of Pharmacy and Medical Sciences, Division of Health Sciences, University of South Australia; Geriatric Medicine Research, Faculty of Medicine, Dalhousie University and Nova Scotia Health Authority, Halifax, NS, Canada College of Pharmacy, Faculty of Health, Dalhousie University<br>Feller, Martin; Institute of Primary Health Care (BIHAM), University of Bern; Department of General Internal Medicine, Inselspital, Bern University Hospital, University of Bern<br>Schneider, Claudio; Department of General Internal Medicine, Inselspital, Bern University Hospital, University of Bern<br>Bhend, Heinz; Hausarzt Praxis Städtli Aarburg<br>Bürki, Pius; Berufsverband der Haus- und Kinderärztinnen Schweiz

	Trelle, S; University of Bern, CTU Bern, University of Bern<br>Spruit, Marco; Department of Information and Computing Sciences, Utrecht University<br>Schwenkglenks, Matthias; Institute of Pharmaceutical Medicine (ECPM), University of Basel; Epidemiology, Biostatistics and Prevention Institute, University of Zurich<br>Rodondi, Nicolas; Institute of Primary Health Care (BIHAM), University of Bern; Department of General Internal Medicine, Inselspital, Bern University Hospital, University of Bern<br>Streit, Sven; Institute of Primary Health Care (BIHAM), University of Bern
Keywords:	Multimorbidity, Polypharmacy, Primary health care

SCHOLARONE™
Manuscripts

Design of 'Optimizing PharmacoTherapy In the Multimorbid Elderly in Primary Care' (OPTICA) to improve medication appropriateness: a cluster randomised controlled trial

Jungo KT[1], Rozsnyai Z[1], Mantelli S[1], Floriani C[1], Löwe AL[1,2], Lindemann F[1], Schwab N[1,2], Meier R[3], Elloumi L[4,5], Huibers CJA[6], Sallevelt BTGM[7], Meulendijk MC[8], Reeve E[9,10], Feller M[1,2], Schneider C[2], Bhend H[11], Bürki P[12], Trelle S[13], Spruit M[4], Schwenkglenks M[14,15], Rodondi N[1,2], Streit S[1]

Affiliations:

[1] Institute of Primary Health Care (BIHAM), University of Bern, Bern, Switzerland

[2] Department of General Internal Medicine, Inselspital, Bern University Hospital, University of Bern, Bern, Switzerland

[3] Institute of Primary Care, University of Zurich, Zurich, Switzerland

[4] Department of Information and Computing Sciences, Utrecht University, Utrecht, The Netherlands

[5] Department of Communication and Cognition, Tilburg University, Tilburg, The Netherlands

[6] Department of Geriatric Medicine, University Medical Centre Utrecht, Utrecht, The Netherlands

[7] Department of Clinical Pharmacy, University Medical Centre Utrecht, Utrecht, The Netherlands

[8] Department of Public Health and Primary Care, Leiden University Medical Center, Leiden, The Netherlands

[9] Quality Use of Medicines and Pharmacy Research Centre, School of Pharmacy and Medical Sciences, Division of Health Sciences, University of South Australia, Adelaide, South Australia, Australia

[10] Geriatric Medicine Research, Faculty of Medicine, Dalhousie University and Nova Scotia Health Authority, Halifax, NS, Canada College of Pharmacy, Faculty of Health, Dalhousie University, NS, Canada

[11] Hausarzt Praxis Städtli Aarburg, Aargau, Switzerland

[12] Berufsverband der Haus- und Kinderärztinnen Schweiz, Bern, Switzerland

[13] CTU Bern, University of Bern, Bern, Switzerland

[14] Institute of Pharmaceutical Medicine (ECPM), University of Basel, Basel, Switzerland

[15] Epidemiology, Biostatistics and Prevention Institute, University of Zurich, Zurich, Switzerland

Corresponding author:

Professor Sven Streit, MD MSc PhD

Sponsor-Investigator

Institute of Primary Health Care (BIHAM), University of Bern, Switzerland

Mittelstrasse 43

3012 Bern, Switzerland

Phone number: +41 31 631 58 75

Email: sven.streit@biham.unibe.ch

Word count: 4835

Key words: Primary Health Care, Multimorbidity, Polypharmacy

For peer review only

ABSTRACT

Introduction:

Multimorbidity and polypharmacy are major risk factors for potentially inappropriate prescribing (over- and underprescribing), and systematic medication reviews are complex and time consuming. In this trial, the investigators aim to determine if a systematic medication review improves medication appropriateness more than standard care in older, multimorbid patients with polypharmacy.

Methods and analysis:

OPTICA (Optimizing PharmacoTherapy In the Multimorbid Elderly in Primary Care) is a cluster randomised, controlled trial that will include outpatients from the Swiss primary care setting, aged ≥65 years with ≥3 chronic medical conditions and concurrent use of ≥5 chronic medications. Patients treated by the same general practitioner constitute a cluster, and clusters are randomised 1:1 to either a standard care sham intervention, in which the general practitioner discusses with the patient if the medication list is complete, or a systematic medication review intervention based on the use of the 'Systematic Tool to Reduce Inappropriate Prescribing'-Assistant (STRIPA). STRIPA is a web-based clinical decision support system that helps customize medication reviews. It is based on the validated 'Screening Tool of Older Person's Prescriptions' (STOPP) and 'Screening Tool to Alert doctors to Right Treatment' (START) criteria to detect potentially inappropriate prescribing. The trial's follow-up period is twelve months. Outcomes will be assessed at baseline, six, and twelve months. The primary endpoint is medication appropriateness, as measured jointly by the change in the Medication Appropriateness Index (MAI) and Assessment of Underutilization (AOU). Secondary endpoints include the degree of polypharmacy, the number of falls and fractures, quality of life, the amount of formal and informal care received by patients, patients' quality adjusted life years, patients' medical costs, cost-effectiveness of the intervention, percentage of recommendations accepted by general practitioners percentage of recommendation rejected by general practitioners, patients' willingness to deprescribe, overprescribing and underprescribing.

Ethics and dissemination:

The ethics committee of the canton of Bern in Switzerland approved the trial protocol. The results of this trial will be published in a peer-reviewed journal.

Main funding:

Swiss National Science Foundation, National Research Programme (NRP 74) "Smarter Healthcare"

Trial registration numbers:

Clinicaltrials.gov: NCT03724539

KOFAM (Swiss national portal): SNCTP000003060

Universal Trial Number: U1111-1226-8013

Protocol version: v1.4, 24.08.2018

For peer review only - http://bmjopen.bmj.com/site/about/guidelines.xhtml

STRENGTHS AND LIMITATIONS

- OPTICA is the first randomised controlled trial to examine the effect of a systematic, structured medication review on medication appropriateness in older, multimorbid patients with polypharmacy in a primary care setting.
- The OPTICA trial shows the usefulness of coding and linking data from electronic medical records and re-using this data to evaluate interventions through a clinical trial.
- The investigators limit selection bias by using screening lists with a random sample of potentially eligible patients and randomising general practitioners after patient recruitment is complete, but cannot eliminate the risk of selection bias.
- The investigators will conduct a sham intervention in accordance with usual care in the control group to improve patient blinding but, by design, they could not blind general practitioners.

INTRODUCTION

Globally, there is a high prevalence of multimorbidity and polypharmacy in people more than 65 years old.[1,2] Multimorbidity is commonly defined as the co-existence of three or more chronic diseases,[3] and polypharmacy is defined as the regular intake of five or more medications.[4] Polypharmacy is often caused by multimorbidity and is linked to a high risk of potentially inappropriate prescribing.[5,6] Appropriate polypharmacy denotes a situation in which "medication use is optimised according to the patients' clinical needs",[7] and inappropriate polypharmacy denotes the use of more medications than clinically indicated. While appropriate polypharmacy can improve quality of life and prevent consequences of disease, inappropriate polypharmacy can harm patients' health.[4] For instance, it can increase the risk of falls and fractures,[8] lead to cognitive decline,[9,10] and it can reduce quality of life.[8] Polypharmacy also increases the risk of drug-drug interactions, drug-disease interactions, and adverse drug events.[11-14] Treatment of older patients with multimorbidity and polypharmacy is a complex problem in primary care and other medical fields and both conditions are increasingly common as populations age. Medication reviews in older adults with multimorbidity and polypharmacy can be complicated and time-consuming.[15] General practitioners (GPs) have reported that time limitations and lack of user-friendly and reliable tools pose significant barriers to regular medication optimization activities in practice.[16]

Evidence suggests that medication review interventions that aim to optimize medication can reduce inappropriate polypharmacy and may improve clinical outcomes.[17] Increasing use of electronic medical records (EMR) and electronic prescribing has opened opportunities to incorporate web-based clinical decision support systems (CDSS) at the point of care. Medication optimization studies that use clinical decision support systems enabled by electronic medical records have shown that the interventions are feasible and acceptable to clinicians, but evidence

For peer review only - http://bmjopen.bmj.com/site/about/guidelines.xhtml

that they lead to more appropriate use of medications and improve clinical outcomes is still limited.[18]

Objectives

The primary aim of the cluster-randomised ‘Optimizing PharmacoTherapy In the Multimorbid Elderly in Primary Care’ (OPTICA) trial is to test a systematic medication review intervention in the Swiss primary care setting and determine if it results in more appropriate use of medications, as measured by the Medication Appropriateness Index (MAI) and Assessment of Underutilization (AOU) than a usual care sham intervention. Secondary outcomes are the degree of polypharmacy, the number of falls and fractures, quality of life, the amount of formal and informal care received by patients, patients’ quality adjusted life years, patients’ medical costs, cost-effectiveness of the intervention, percentage of recommendations accepted and rejected by GPs, patients’ willingness to deprescribe, overprescribing and underprescribing.

METHODS AND ANALYSIS

General study design and setting

The OPTICA trial is a cluster randomised controlled trial (RCT), coordinated at the Institute of Primary Health Care of the University of Bern (BIHAM). Participating GPs, who will each systematically recruit multimorbid, older patients with polypharmacy, define the clusters. Through simple randomisation, the GPs will be allocated to the structured medication review or a usual care based sham intervention.

The investigators used the SPIRIT checklist when they wrote this protocol.[19] Please refer to Figure 1 for the study flow chart and Figure 2 for a description of data flow within the OPTICA trial.

Cluster definition

The trial will be conducted in about 40 primary care offices in the German-speaking regions of Switzerland. Each participating GP, who prescribes the medication of their patients, constitutes a cluster. GPs were recruited from October 2017 until June 2018 from the pool of teaching physicians at the University of Bern and from the group of GPs who attended project presentations hosted by the BIHAM throughout 2017 and 2018. All eligible GPs who showed interest in the OPTICA trial were visited by the investigators at their GP office and given a detailed explanation of the trial.[20] As of March 2019, 85% of the participating GPs are male. Eighty-two percent of the GPs work in group practice, while 18% work in single practice. Forty-nine percent of GPs work in the countryside and 51% work in urban and sub-urban areas.

For peer review only - http://bmjopen.bmj.com/site/about/guidelines.xhtml
For peer review only

Randomisation

Randomisation is done after the cluster has been completed. The randomisation is done centrally in a web-based system (REDCap) by a study team member after all cluster information has been entered. Each participating GP is allocated 1:1 to the intervention group or the control group, using unstratified randomisation with a random sequence of block sizes of two and four. An independent statistician, who is otherwise not involved in the trial, generated the randomisation list. To uphold the concealment of allocation only system administrators who are otherwise not involved in the trial can access the randomisation list.

Eligibility criteria

Inclusion criteria

General practitioner level: To be eligible for participation, GPs must be participating in the "Family medicine ICPC Research using Electronic medical records" (FIRE) project led by the Institute of Primary Care of the University of Zurich (IHAMZ).[21] The electronic medical records (EMR) they use in their GP office have to be compatible with the FIRE database, which is a database with anonymous data from the EMR of more than 500 GPs in Switzerland. The database of the FIRE project contains administrative data, vital data, laboratory values, International Classification of Primary Care 2 (ICPC-2) codes for diagnoses and information on medications prescribed. Participating GPs can, only for the purpose of this trial, identify individual patients in the usually anonymous FIRE database and use their data. All participating GPs must complete an online training for Good Clinical Practice (GCP).

Patient level: Patients must be enlisted by one of the participating GPs, so that this GP is their main prescribing physician and they must see their GP regularly. The investigators left the definition of regularity to the judgment of participating physicians. Patients must be ≥65 years of age, have ≥3 chronic diseases based on ICPC-2 coding or based on the GP's clinical decision (multimorbidity), and regularly take ≥5 medications (polypharmacy). Written informed consent must be sought from patients or, if patients suffer from cognitive impairment, their relative prior to enrolment. This consent includes the right to obtain data from the FIRE project about the study participant.

Exclusion criteria

General practitioners level: In group practices, only one GP can take part in the trial.

Patient level: To maximize the generalizability of the study population, the investigators kept exclusion criteria to a minimum. They excluded patients currently taking part in another interventional trial.

Intervention

For peer review only

The intervention, a systematic medication review, includes the use of a web-based clinical decision support system called ‘Systematic Tool to Reduce Inappropriate Prescribing’- Assistant (STRIPA), developed by a team from the Utrecht University and the University Medical Center Utrecht in the Netherlands.[22] STRIPA is based on all algorithms of the ‘Screening Tool of Older Person’s Prescriptions’ (STOPP) and ‘Screening Tool to Alert doctors to Right Treatment’ (START) criteria (version 2),[23] which are expert-consensus lists of inappropriate and appropriate medications for older adults that consider co-existing medical conditions and are thus suited for optimizing the prescriptions of multimorbid, older patients.[24] STRIPA combines implicit and explicit prescribing tools and takes a stepped approach that actively encourages patient involvement in decision-making.[22,25] Taking into account medications, diagnoses, laboratory values, and vital data, the STRIPA generates recommendations for physicians about “underprescribing, ineffective prescribing, overprescribing, side effects, contraindications, (...) drug-drug and drug-disease interactions, incorrect dosages/dosing frequencies, and practical intake issues”.[22] The intervention’s recommendations allow patients and GPs to conduct a shared decision-making process about the patients’ medication intake, so patient preferences play an important role in this trial.

A STRIPA validation study with GPs based on two test cases showed appropriate decisions increased and inappropriate decisions decreased during a medication review of an older, multimorbid patient with polypharmacy.[25] STRIPA is being tested in the European multicentre clinical trial ‘OPtimising thERapy to prevent Avoidable hospital admissions in Multimorbid older people’ (OPERAM) in the hospital setting to find out if it can reduce drug-related hospital re-admissions.[26]

This study adapted the intervention from the OPERAM trial so it could be tested in the primary care setting.[27] The intervention comprises six steps:

1. This data will be imported from the FIRE database into the STRIPA: patient’s baseline characteristics (e.g., sex, age); vital data and laboratory measurements (e.g., kidney function, blood pressure), patients’ signs and symptoms, different scores (e.g., HASBLED), ICPC-2 coded diagnoses, and medications entered as Anatomical Therapeutic Chemical (ATC) codes.

2. GPs log in to the ‘Systematic Tool to Reduce Inappropriate Prescribing’- Assistant (STRIPA) and see a list with their recruited patients’ identifiers. When they click on a patient’s ID, the information exported from the EMR to STRIPA via FIRE becomes visible (from Step 1). The GP can add unrecorded values or adjust values (e.g., when more recent laboratory values are available). This might be necessary because it might take some time to obtain the FIRE data and to import it to STRIPA and hence the information might change in the meantime.

3. Use a drag and drop function to link each medication to a diagnosis.

For peer review only - http://bmjopen.bmj.com/site/about/guidelines.xhtml
For peer review only

4. Run the analysis in STRIPA and then look at STRIPA's recommendations. After the analysis is finished, STRIPA records the recommendations it generates so they will be available for later analysis and generates a PDF report that can be saved by GPs.
5. GPs then must decide if they agree with STRIPA's advice given and if they will present it to their patient. They can follow, partly follow, or decline STRIPA's recommendations.
6. At the next appointment with the same patient, the GP will make the recommendations that they consider appropriate. GP and patient will decide together, in a shared decision-making process, which recommendations to implement. GPs will work from a checklist that explains how to conduct the shared decision-making process so that the patient's preferences are met. The shared-decision making process is based on Elwyn et al.'s model[28] and was adapted from the OPERAM trial. The four key elements of this shared decision-making process are "choice talk", "option talk", "preference talk", and "decision talk".[28] Patients may follow, partly follow or decline the recommendations. Because multimorbid patients are likely to have multiple prescribers, GPs and patients can discuss recommendations with other prescribers, e.g., specialists. The GP records in the REDCap study database the patient's decisions and the reasons recommendations were accepted, partially accepted, or declined. These records will be analysed later.

Before the intervention, GPs in the intervention group will watch an instruction video and read training material that will guide them through the intervention step-by-step.

Sham intervention

Patients assigned to the control arm receive a sham intervention from their GP — a medication discussion in accordance with usual care. This means that the GP will, as usual in standard care, ask the patient if the current list of medication is complete or needs to be adapted, then share decision-making with the patient about possible changes. The GPs who do the sham intervention will receive the same shared decision-making checklist as the GPs in the intervention group. The investigators explicitly ask GPs not to deviate from their usual practice when they review the patient's medication and not to use additional tools. After the sham intervention, GPs record procedures, decisions, and reasons in the REDCap study database for later analysis. This sham intervention ensures that the medication list of control group patients, which will be used to assess study outcomes, is up-to-date, while also ensuring patients are blinded.

Screening and enrolment

For patient recruitment, the investigators use data from the FIRE project to prepare a screening list for each GP that contains a random selection of eligible patients. On each screening list, there are about 20 patients who were randomly sampled from all enlisted patients after they met the age criteria and were determined to regularly take at least five chronic medications, based on the

For peer review only - http://bmjopen.bmj.com/site/about/guidelines.xhtml

Pharmacy-based Cost Group (PCG) model.[29-31] Since ICPC-2 coding is suboptimal in many GP offices, the investigators decided not to include the chronic disease criterion when they prepare the screening lists. From the list, GPs systematically recruit eight to ten patients who meet the full inclusion criteria, which now include the number of chronic diseases, and do not meet any of the exclusion criteria. GPs will then recruit patients starting either at the bottom of the list or the top. If GPs have finished the first screening list without recruiting the minimum amount of participants, the investigators will provide them with a second list.

Since the screening lists are compiled from a random patient sample from each GP office based on FIRE data, patients on the list may have been treated by a different GP who works in the same group practice. It is also possible that patients on the list changed their GP or have died. Since prescriptions frequently change[32], some patients with polypharmacy might not have qualified for the screening list when data was last exported. For all these reasons, the protocol allows GPs to skip patients on the screening list if they provide an explanation, or to recruit patients who are not on the list but fulfil the inclusion and do not meet the exclusion criteria. After identifying patients on the list and verifying their eligibility, GPs then inform the patient about the study and seek their consent. Study-related appointments are added to the patients' regular appointments at no charge.

Data collection

Study participants or their relatives are followed up by phone at baseline and at six and twelve months after study enrolment to complete data collection. At these times, complementary information from the FIRE database, including information about medications and chronic diseases, is imported into the REDCap study database.[21]

Blinding procedures

The OPTICA trial is blinded to the extent the cluster design of this randomised controlled trial allows. Our method of partial blinding, which is similar to that used in the OPERAM trial,[26] is set out in detail in Table 1.

Table 1: Blinding status and measures to assure blinding

Role	Blinding status	How to achieve blinding
General practitioners	Unblinded	When screening for patients and seeking informed consent, GPs are still blinded, as each cluster (GP) will be only randomised after the cluster is full (8-10 patients). The screening list GPs use for recruitment contains a random sample of potentially eligible patients, which was generated from FIRE data by a blinded study team member. All this is done to prevent selection bias

For peer review only - http://bmjopen.bmj.com/site/about/guidelines.xhtml

		However, our study design makes it impossible to blind GPs throughout the trial. When GPs do the medication review/discussion, they know what group they are allocated to. Nevertheless, GPs in the control arm do not know the procedures in the intervention arm, which prevents cross-contamination; if they knew the procedure in the intervention arm, they might adapt their usual care.
Data collectors and assessors	Blinded	The randomisation of GPs is kept concealed from the team that makes follow-up calls to avoid interviewer bias. Data collectors and assessors have no access to unblinded study information in the database or to local source data. If a serious adverse event (SAE) occurs, the study coordinator and project manager will be informed.
Data manager and data analysist	Unblinded	The investigators cannot blind our data managers and analysts because they can see the differences in data structure between the study groups. This is why the investigators use a new data analyst to prepare a clean data set with truncated data to conduct a blinded analysis of the primary outcome.
Study coordinator and project manager, including principal investigator	Unblinded	The study coordinator and project manager the OPTICA trial know the treatment allocation. They are responsible for collecting information about SAEs and performing safety assessments.
Patients	Partially blinded	Patients stay partially blinded. They are only given a "high-level description" of the study question so they know that their GP has been allocated to one of two study groups, but they do not know which one. To uphold patient blinding, patients in the control group will meet their GP for a medication discussion and a shared decision-making about their prescriptions. This means patients in each study arm see GPs the same number of times during the trial and cannot guess their allocation status based on the number of consultations.
The OPTICA trial's approach to blinding resembles the approach used in the OPERAM trial.[26]		

For peer review only

Follow-up

Outcome information is collected at baseline, at six, and at twelve months via telephone calls with patients or relatives conducted by a blinded study team member, and through FIRE database exports. The study team will make every reasonable effort to keep each patient in the study until all planned treatments and assessments have been performed. But patients may withdraw from the study or be withdrawn when they are lost to follow-up.

Assessment of primary outcome

The primary endpoint is medication appropriateness, measured jointly by the Medication Appropriateness Index (MAI), which assess overprescribing, and the Assessment of Underutilization (AOU), which assesses overprescribing, in each study group.[33-35] Blinded study

For peer review only - http://bmjopen.bmj.com/site/about/guidelines.xhtml

team members assess the MAI for each regular medication study participants take and assess the AOU for each chronic condition study participants have. The investigators will use the 10-item version of the MAI; the score for each medication ranges from 0 to 18.[35] For the AOU, assessors decide for each chronic condition if there is i) no omission, ii) marginal omission, or iii) omission of indicated medication.[36-38] For at least 32 cases (10% of the targeted sample size), the investigators will conduct a blinded independent double assessment of the MAI and the AOU to check inter-rater reliability. The investigators will import information about diagnoses (coded in ICPC-2) and medication intake from the FIRE database into the study database and use it for these assessments.

Assessment of secondary outcomes

The following secondary outcomes are assessed at baseline, six, and twelve months:

- Degree of polypharmacy; i.e., the number of regular long-term medications patients take
- Number of falls and fractures in the last six months
- Quality of life, based on the 5-level version of the European Quality of Life-5 Dimensions questionnaire (EQ-5D), including pain/discomfort[39,40]
- Amount of formal care received: number and length of stay of planned and unplanned hospitalizations; visits to the emergency room without inpatient hospitalization; GP visits; medical specialist visits (differentiated by specialty); hospital outpatient visits; inpatient stays; length of stay at rehabilitation facilities; physiotherapist and other allied therapist visits; nursing home admissions (in patients who were living in the community at baseline); length of stay in nursing homes; and, number of home nursing visits
- Amount of informal care received: unpaid care by, e.g., family members, relatives, friends
- Quality-adjusted life years (QALYs) accrued in one year,[41] measured as a function of the length and the quality of life
- Direct medical costs accrued in one year, measured by combining formal and informal healthcare resource use observed in the trial, including the time GPs spend on the intervention and software costs, and Swiss unit costs from sources external to the trial
- Cost-effectiveness of the intervention, calculated by combining clinical data, quality of life data, and healthcare use data collected in the trial
- Overprescribing, measured by the Medication Appropriateness Index (MAI)
- Underprescribing, measured by the Assessment of Underutilization (AOU)

The following outcome will be assessed after the intervention:

- Percentage of STRIPA recommendations accepted and rejected by GPs

The following secondary outcome is assessed at baseline only:

- Patients' willingness to deprescribe, measured with the validated 'revised Patient Attitudes Towards Deprescribing' (rPATD) questionnaire[42,43]

For peer review only - http://bmjopen.bmj.com/site/about/guidelines.xhtml

Safety outcomes include adverse events, serious adverse events and device deficiencies.

Study duration

GPs can recruit the patients over a period of at least six months, so they can integrate recruitment into their daily practice. Recruited patients are followed up for one year.

Sample size

Sample size calculation is based on testing the two co-primary outcomes for superiority and uses the Bonferroni-approach to account for multiple testing. The investigators assumed that 35% of patients in the control group and 60% of patients in the intervention group will improve their total Medication Appropriateness Index (MAI) score (at least one less point) and that 10% of patients in the control group and 30% of patients in the intervention group will have a better Assessment of Underutilization (AOU) score (at least one fewer prescribing omission). Intra-cluster correlations (ICC) of 0.01 to 0.05 are typically found for binary outcomes in elderly individuals.[44] The investigators conservatively assumed an ICC of 0.05 to calculate sample size. The investigators fixed the type I error at a Bonferroni-corrected two-sided alpha level of 0.025.

Based on a two-sample comparison of proportions and a pre-specified number of clusters of about 40 (about 20 per arm), seven patients per cluster are required to detect a difference of 20% between the two groups in the proportion of improvement in the MAI, with a power of 90%. Using the same assumption for the AOU, the investigators found they also need seven patients per cluster to detect a difference of 20%. The investigators thus need a sample of 280 patients (140 per arm) to provide 90% power to detect a significant improvement in both the MAI score and the AOU. To account for attrition from dropout or death (15% estimated), the number of patients per cluster was increased to 8 (max. 10), so the final sample size should be about 320 patients (160 per arm). The investigators will closely support GPs to help them reach our target sample size.

Statistical analysis

In case data on outcomes are incomplete, the investigators will use multiple imputation to replace missing values, taking data clustering into account. No interim analyses are planned.

There are two co-primary outcomes: improvement in the MAI at twelve months, defined as decrease of at least one point, and improvement in the AOU at twelve months, defined as least one less prescribing omission. Both outcomes will be tested separately; success is indicated by the significance of at least one of the two tests.

For the co-primary outcomes, the investigators will present and compare the proportion of patients whose MAI and AOU score improved in the control and intervention group. The relative difference between groups will be determined in a mixed-effects logistic model with a random intercept at

For peer review only

the GP level to account for clustering. The effect measure for the primary outcomes will be odds ratios. The relative difference will be presented as odds ratio with a 95% confidence interval. The primary analysis will be an intention-to-treat (ITT) analysis. In a per-protocol (PP) analysis, the investigators will only evaluate patients who adhered to the protocol and exclude patients who violate any inclusion criteria.

Secondary binary outcomes will be evaluated like the primary outcomes. Secondary continuous outcomes will be analysed using random-effects linear regression with a random intercept at the GP level. Models will also be adjusted for the baseline value as a covariate. Secondary count outcomes will be analysed using random-effects Poisson regression with a random intercept at the GP level.

Health economic analyses will assess i) resource use and cost differences between the trial arms, ii) differences in quality-adjusted lifetime between the trial arms, expressed as quality-adjusted life years [QALYs], and iii) a comparison of cost-effectiveness between intervention and standard care.

Because cluster-randomisation may not balance characteristics between groups to match individual-level randomisation, the investigators will adjust each model for patient- and physician-level variables to account for case-mix differences between groups and potential recruitment bias in a sensitivity analysis. They will also account for the correlated nature of data among GPs by using multilevel mixed-effects models. Unadjusted models will be provided for information only.

PATIENT AND PUBLIC INVOLVEMENT

Multimorbid, older patients with polypharmacy are represented in the independent Safety and Data Monitoring Board of the OPTICA trial. Throughout the trial, the Safety and Data Monitoring Board, which will consist of one GP, one statistician, and one multimorbid, older layperson with polypharmacy, will meet regularly to discuss safety and data management issues. Patients are not actively involved in recruiting study participants, but play a key role in shared decision-making during the intervention and sham intervention. The investigators have created a priority list of questionnaire components to reduce the burden of the intervention on very old and sick study participants by reducing the duration of the follow-up calls where necessary. At the end of the study, the investigators will disseminate the results to study participants in a letter.

DISCUSSION

This protocol paper highlights the features of the OPTICA trial, the first RCT to test an intervention based on the STRIPA clinical decision support system, which helps GPs customize medication reviews and optimize polypharmacy in primary care.

This clinical trial compares the effect of a structured medication review on medication appropriateness in a Swiss primary care setting and compares it to a sham intervention.

For peer review only - http://bmjopen.bmj.com/site/about/guidelines.xhtml

Systematic medication review may facilitate shared decision-making and medication appropriateness, especially for GPs who treat complex multimorbid patients with polypharmacy. It may also improve patients' quality of life and health economic outcomes.

OPTICA is subject to the following limitations:

- Despite taking precautions to avoid and reduce selection bias (cluster randomisation to avoid learning effect, screening lists for patient recruitment, randomisation of GPs after patient recruitment), selection bias is still possible because of the study design. Patients who are more engaged and/or more likely to adhere to advice may be more likely to be enrolled in the trial, which might decrease the representativeness of the study population and the generalizability of our results. The investigators will use data from FIRE to compare the characteristics of study participants with those of non-participants.
- The investigators chose a usual care sham intervention in the control group to improve patient blinding, but this design does not eliminate the risk GPs in the control group will be contaminated by the thematic of the trial (risk of deviating from their previous routine prescribing practices). This sham intervention also might lead GPs to suggest medication changes that could improve the appropriateness of patients' medications.
- Outcome assessment is based on self-reported data from patients and relatives, and on data from FIRE, so some events may be missed. To ensure FIRE data is as complete as possible, GPs must code their patient's medication intake and their diagnoses correctly. Diagnoses require ICPC-2 codes. GPs were instructed, in face-to-face meetings, about how to code and update data in their EMR for patients in this trial.[20]
- Like all interventions that take an eHealth approach (STRIPA in this case), people have different perceptions of what is user-friendly. New procedures may seem complicated in comparison to usual care. The investigators tried to simplify the process by i) using EMR data so that GPs do not have to enter all the data themselves, but only update them if needed; and ii) giving detailed instructions for using STRIPA in writing and in an online video training.
- It may be difficult to follow-up multimorbid, older patients, who are often excluded from trials[45]. The investigators believe that the task is worth the effort because trial results need to be generalizable to exactly this population. To reduce the duration of the phone calls with particularly weak study participants only the core elements of the study questionnaire will be used, if necessary, based on a predefined priority list.

The OPTICA trial has the following strengths:

- The OPTICA trial is the first RCT to examine the effect of STRIPA on medication appropriateness in older, multimorbid patients with polypharmacy in a primary care setting.
- OPTICA is the first RCT to test the use of software-based structured medication review in Swiss primary care.

For peer review only - http://bmjopen.bmj.com/site/about/guidelines.xhtml

- OPTICA demonstrates the usefulness of coding and linking data from EMR, and re-using this data to evaluate interventions through a clinical trial.
- In the OPTICA trial, the investigators do not exclude patients with cognitive impairment if a relative gives their informed consent, because patients with cognitive impairment are especially prone to polypharmacy, yet they have been excluded from many other trials.

Conclusion

The OPTICA trial will compare the effect of the systematic medication review that uses STRIPA and medication discussions between patients and GPs to usual care (sham intervention) with the goal of improving medication appropriateness. The investigators expect the intervention to improve the quality of life and health status of a rapidly aging population with increasing multimorbidity and polypharmacy. The study results will inform other studies and interventions designed to optimize medication use by integrating a clinical decision support system with electronic medical records.

CURRENT STATUS OF THE OPTICA TRIAL

The OPTICA trial began recruiting December 2018. By early April 2019, 213 patients (about 66% of the target sample size) have been recruited. The investigators have randomised 16 out of about 40 GPs. Last patient out is expected in the second half of 2020.

ETHICS

The ethics committee of the canton of Bern (Switzerland) and the Swiss regulatory authority (Swissmedic) approved the study protocol and other documentation on study conduct (BASEC ID: 2018-00914). The ethics committee and Swissmedic will receive annual safety reports and information about study stops/end and protocol amendments, as per local requirements.

All participant data will be handled according to the principles of the Declaration of Helsinki.[46] The OPTICA trial complies with all applicable standards of the ICH-GCP guideline.[47]

Data management, monitoring, safety reporting and audits meet the requirements of Swiss law. The investigators uphold the principle of patients' right to privacy and comply with applicable privacy laws. Confidentiality and anonymity of the patients shall be guaranteed when the data is presented at scientific meetings or published in scientific journals. Only selected study team members will have access to the final trial dataset.

Risks, including human failure and software malfunction, cannot be excluded. But STRIPA only makes prescription recommendations to GPs in the intervention group. Participating GPs are experienced (mean age of experience as GP: 16 years), and will take the final decision about whether to accept the recommendations and to present them to the patient. Patients thus are not

For peer review only

exposed to more risk than they would be in standard care. This clinical trial should entail minimal risk for participants and the benefit-risk ratio is positive.

Basler Versicherungen will provide insurance and cover eventual damages. Participating GPs have signed a non-disclosure agreement.

DISSEMINATION

OPTICA embraces an open access policy and will vigorously disseminate all of all resulting data, study results, and publications. No data are currently available. The investigators closely collaborate with National Research Programme 74 (NRP74) to optimise dissemination of the study results to the public.

AUTHOR CONTRIBUTIONS

Authorship eligibility is based on the four ICMJE authorship criteria.

Study concept and design: Trelle, Spruit, Schwenkglenks, Jungo, Bhend, Meier, Rodondi, Streit

Drafting of the manuscript: Jungo, Streit

Critical contribution to the study protocol and revision of the manuscript for important intellectual content: Mantelli, Floriani, Löwe, Lindemann, Rozsnyai, Schwab, Meier, Sallevelt, Huibers, Meulendijk, Elloumi, Schneider, Feller, Bhend, Reeve, Bürki, Trelle, Spruit, Schwenkglenks, Rodondi

Statistical analysis: Trelle

Obtained funding: Rodondi, Streit

Administrative, technical, or material support: Schwab

Supervision: Streit

FUNDING STATEMENT

This work is supported by the Swiss National Science Foundation, within the framework of the National Research Programme 74 (NRP74) under contract number 407440_167465 (to Prof. Sven Streit and Prof. Nicolas Rodondi).

COMPETING INTERESTS STATEMENT

The authors state that they have no competing interest with regard to the intellectual concern and proprietary affairs.

CONVEYANCE AND PEER REVIEW

For peer review only - http://bmjopen.bmj.com/site/about/guidelines.xhtml

The trial protocol has been peer-reviewed for ethical, regulatory, and funding approval before submission.

ACKNOWLEDGEMENT

The entire OPTICA study team would like to acknowledge the enormous help provided by all general practitioners participating in OPTICA and for their efforts to recruit their older multimorbid patients with polypharmacy. They also thank Kali Tal for her editorial suggestions.

For peer review only

For peer review only - http://bmjopen.bmj.com/site/about/guidelines.xhtml

REFERENCES

1. Violan C, Foguet-Boreu Q, Flores-Mateo G, et al. Prevalence, determinants and patterns of multimorbidity in primary care: a systematic review of observational studies. *PloS one* 2014;9(7):e102149. doi: 10.1371/journal.pone.0102149 [published Online First: 2014/07/23]
2. Rawle MJ, Richards M, Davis D, et al. The prevalence and determinants of polypharmacy at age 69: a British birth cohort study. *BMC geriatrics* 2018;18(1):118. doi: 10.1186/s12877-018-0795-2 [published Online First: 2018/05/18]
3. Harrison C, Britt H, Miller G, et al. Examining different measures of multimorbidity, using a large prospective cross-sectional study in Australian general practice. *BMJ open* 2014;4(7):e004694. doi: 10.1136/bmjopen-2013-004694 [published Online First: 2014/07/13]
4. Gnjidic D, Hilmer SN, Blyth FM, et al. Polypharmacy cutoff and outcomes: five or more medicines were used to identify community-dwelling older men at risk of different adverse outcomes. *Journal of clinical epidemiology* 2012;65(9):989-95. doi: 10.1016/j.jclinepi.2012.02.018 [published Online First: 2012/06/30]
5. Mannucci PM, Nobili A. Multimorbidity and polypharmacy in the elderly: lessons from REPOSI. *Internal and emergency medicine* 2014;9(7):723-34. doi: 10.1007/s11739-014-1124-1 [published Online First: 2014/08/29]
6. Naples JG, Hajjar ER. Multimorbidity and Polypharmacy. In: Stegemann S, ed. Developing Drug Products in an Aging Society: From Concept to Prescribing. Cham: Springer International Publishing 2016:549-61.
7. Cadogan CA, Ryan C, Hughes CM. Appropriate Polypharmacy and Medicine Safety: When Many is not Too Many. *Drug Saf* 2016;39(2):109-16. doi: 10.1007/s40264-015-0378-5 [published Online First: 2015/12/23]
8. Pan H-H, Li C-Y, Chen T-J, et al. Association of polypharmacy with fall-related fractures in older Taiwanese people: age- and gender-specific analyses. *BMJ open* 2014;4(3):e004428. doi: 10.1136/bmjopen-2013-004428
9. Niikawa H, Okamura T, Ito K, et al. Association between polypharmacy and cognitive impairment in an elderly Japanese population residing in an urban community. *Geriatrics & gerontology international* 2017;17(9):1286-93. doi: 10.1111/ggi.12862 [published Online First: 2016/09/16]
10. Oyarzun-Gonzalez XA, Taylor KC, Myers SR, et al. Cognitive Decline and Polypharmacy in an Elderly Population. *Journal of the American Geriatrics Society* 2015;63(2):397-99. doi: doi:10.1111/jgs.13283
11. Lavan AH, Gallagher P. Predicting risk of adverse drug reactions in older adults. *Therapeutic advances in drug safety* 2016;7(1):11-22. doi: 10.1177/2042098615615472 [published Online First: 2016/02/03]
12. Castilho ECD, Reis AMM, Borges TL, et al. Potential drug-drug interactions and polypharmacy in institutionalized elderly patients in a public hospital in Brazil. *Journal of psychiatric and mental health nursing* 2018;25(1):3-13. doi: 10.1111/jpm.12431 [published Online First: 2017/09/12]
13. Rosholm JU, Bjerrum L, Hallas J, et al. Polypharmacy and the risk of drug-drug interactions among Danish elderly. A prescription database study. *Danish medical bulletin* 1998;45(2):210-3. [published Online First: 1998/05/20]
14. Maher RL, Hanlon J, Hajjar ER. Clinical consequences of polypharmacy in elderly. *Expert Opinion on Drug Safety* 2014;13(1):57-65. doi: 10.1517/14740338.2013.827660
15. Tarn DM, Paterniti DA, Kravitz RL, et al. How Do Physicians Conduct Medication Reviews? *Journal of General Internal Medicine* 2009;24(12):1296. doi: 10.1007/s11606-009-1132-4
16. Mantelli S, Jungo KT, Rozsnyai Z, et al. How general practitioners would deprescribe in frail oldest-old with polypharmacy - the LESS study. *BMC Fam Pract* 2018;19(1):169. doi: 10.1186/s12875-018-0856-9 [published Online First: 2018/10/14]
17. Huiskes VJB, Burger DM, van den Ende CHM, et al. Effectiveness of medication review: a systematic review and meta-analysis of randomized controlled trials. *BMC Family Practice* 2017;18(1):5. doi: 10.1186/s12875-016-0577-x
18. Scott IA, Pillans PI, Barras M, et al. Using EMR-enabled computerized decision support systems to reduce prescribing of potentially inappropriate medications: a narrative review. *Therapeutic advances in drug safety* 2018;9(9):559-73. doi: 10.1177/2042098618784809
19. Chan AW, Tetzlaff JM, Altman DG, et al. SPIRIT 2013 statement: defining standard protocol items for clinical trials. *Annals of internal medicine* 2013;158(3):200-7. doi: 10.7326/0003-4819-158-3-201302050-00583 [published Online First: 2013/01/09]
20. Jungo K, Löwe A, Mantelli S, et al. Klinische Studie zur Medikamentenoptimierung bei älteren Patient /-innen mit Polypharmazie: Die OPTICA-Studie. *Hospital and Primary Care* 2018;18(8):100–02.

For peer review only - http://bmjopen.bmj.com/site/about/guidelines.xhtml

21. Chmiel C, Bhend H, Senn O, et al. The FIRE project: a milestone for research in primary care in Switzerland. *Swiss medical weekly* 2011;140:w13142. doi: 10.4414/smw.2011.13142 [published Online First: 2011/02/01]
22. Drenth-van Maanen AC, Leendertse AJ, Jansen PAF, et al. The Systematic Tool to Reduce Inappropriate Prescribing (STRIP): Combining implicit and explicit prescribing tools to improve appropriate prescribing. *Journal of evaluation in clinical practice* 2018;24(2):317-22. doi: 10.1111/jep.12787 [published Online First: 2017/08/05]
23. Huibers CJA, Sallevelt BTGM, de Groot DA, et al. Conversion of STOPP/START version 2 into coded algorithms for software implementation: A multidisciplinary consensus procedure. *International Journal of Medical Informatics* 2019;125:110-17. doi: doi.org/10.1016/j.ijmedinf.2018.12.010
24. O'Mahony D, O'Sullivan D, Byrne S, et al. STOPP/START criteria for potentially inappropriate prescribing in older people: version 2. *Age and ageing* 2015;44(2):213-18. doi: 10.1093/ageing/afu145
25. Meulendijk MC, Spruit MR, Drenth-van Maanen AC, et al. Computerized Decision Support Improves Medication Review Effectiveness: An Experiment Evaluating the STRIP Assistant's Usability. *Drugs & Aging* 2015;32(6):495-503. doi: 10.1007/s40266-015-0270-0
26. Adam L, Moutzouri E, Loewe A, et al. Rationale and design of OPtimising thERapy to prevent Avoidable hospital admissions in Multimorbid older people (OPERAM): a cluster randomised controlled trial. submitted for publication, 2019.
27. Cowley E, Murphy K, Spruit M, et al. Intervention protocol: Optimising thERapy to prevent Avoidable hospital admission in the Multi-morbid elderly (OPERAM): a structured medication review with support of a computerised decision support system *submitted for publication* 2019
28. Elwyn G, Frosch D, Thomson R, et al. Shared decision making: a model for clinical practice. *J Gen Intern Med* 2012;27(10):1361-7. doi: 10.1007/s11606-012-2077-6 [published Online First: 2012/05/24]
29. Lamers LM. Risk-adjusted capitation payments: developing a diagnostic cost groups classification for the Dutch situation. *Health policy (Amsterdam, Netherlands)* 1998;45(1):15-32. [published Online First: 1998/06/06]
30. Lamers LM, van Vliet RC. The Pharmacy-based Cost Group model: validating and adjusting the classification of medications for chronic conditions to the Dutch situation. *Health policy (Amsterdam, Netherlands)* 2004;68(1):113-21. doi: 10.1016/j.healthpol.2003.09.001 [published Online First: 2004/03/23]
31. Lamers LM, Vliet RC. Health-based risk adjustment Improving the pharmacy-based cost group model to reduce gaming possibilities. *The European journal of health economics : HEPAC : health economics in prevention and care* 2003;4(2):107-14. doi: 10.1007/s10198-002-0159-9 [published Online First: 2004/12/21]
32. von Buedingen F, Hammer MS, Meid AD, et al. Changes in prescribed medicines in older patients with multimorbidity and polypharmacy in general practice. *BMC Family Practice* 2018;19(1):131. doi: 10.1186/s12875-018-0825-3
33. Hanlon JT, Schmader KE, Ruby CM, et al. Suboptimal prescribing in older inpatients and outpatients. *J Am Geriatr Soc* 2001;49(2):200-9. [published Online First: 2001/02/24]
34. Hanlon JT, Schmader KE, Samsa GP, et al. A method for assessing drug therapy appropriateness. *Journal of clinical epidemiology* 1992;45(10):1045-51. [published Online First: 1992/10/01]
35. Samsa GP, Hanlon JT, Schmader KE, et al. A summated score for the medication appropriateness index: development and assessment of clinimetric properties including content validity. *Journal of clinical epidemiology* 1994;47(8):891-6. [published Online First: 1994/08/01]
36. Dove S, Ruby C, Hanlon J, et al. The effect of hospitalization on suboptimal prescribing in elderly patients. American Society of Consultant Pharmacists 27th Annual Meeting and Exhibition. Nashville, Tenn, 1996.
37. Jeffery S, Ruby C, Hanlon J, et al. The impact of an interdisciplinary team on suboptimal prescribing in a long term care facility. *Consult Pharm* 1999;14(21):1386–91.
38. Wright RM, Sloane R, Pieper CF, et al. Underuse of indicated medications among physically frail older US veterans at the time of hospital discharge: results of a cross-sectional analysis of data from the Geriatric Evaluation and Management Drug Study. *The American journal of geriatric pharmacotherapy* 2009;7(5):271-80. doi: 10.1016/j.amjopharm.2009.11.002 [published Online First: 2009/12/02]
39. Janssen MF, Pickard AS, Golicki D, et al. Measurement properties of the EQ-5D-5L compared to the EQ-5D-3L across eight patient groups: a multi-country study. *Quality of life research : an international journal of quality of life aspects of treatment, care and rehabilitation* 2013;22(7):1717-27. doi: 10.1007/s11136-012-0322-4 [published Online First: 2012/11/28]
40. EuroQol--a new facility for the measurement of health-related quality of life. *Health policy (Amsterdam, Netherlands)* 1990;16(3):199-208. [published Online First: 1990/11/05]

For peer review only - http://bmjopen.bmj.com/site/about/guidelines.xhtml

41. Weinstein MC, Torrance G, McGuire A. QALYs: the basics. *Value in health : the journal of the International Society for Pharmacoeconomics and Outcomes Research* 2009;12 Suppl 1:S5-9. doi: 10.1111/j.1524-4733.2009.00515.x [published Online First: 2009/03/11]
42. Reeve E, Anthony AC, Kouladjian O'Donnell L, et al. Development and pilot testing of the revised Patients' Attitudes Towards Deprescribing questionnaire for people with cognitive impairment. *Australasian journal on ageing* 2018;37(4):E150-e54. doi: 10.1111/ajag.12576 [published Online First: 2018/08/08]
43. Reeve E, Low LF, Shakib S, et al. Development and Validation of the Revised Patients' Attitudes Towards Deprescribing (rPATD) Questionnaire: Versions for Older Adults and Caregivers. *Drugs Aging* 2016;33(12):913-28. doi: 10.1007/s40266-016-0410-1 [published Online First: 2016/10/28]
44. Smeeth L, Ng ES. Intraclass correlation coefficients for cluster randomized trials in primary care: data from the MRC Trial of the Assessment and Management of Older People in the Community. *Controlled clinical trials* 2002;23(4):409-21. [published Online First: 2002/08/06]
45. Buffel du Vaure C, Dechartres A, Battin C, et al. Exclusion of patients with concomitant chronic conditions in ongoing randomised controlled trials targeting 10 common chronic conditions and registered at ClinicalTrials.gov: a systematic review of registration details. *BMJ open* 2016;6(9):e012265. doi: 10.1136/bmjopen-2016-012265
46. World Medical Association Declaration of Helsinki: ethical principles for medical research involving human subjects. *The Journal of the American College of Dentists* 2014;81(3):14-8. [published Online First: 2015/05/09]
47. Guideline for Good Clinical Practice E6(R1): International Council on Harmonisation of Technical Requirements for Registration of Pharmaceuticals for Human Use (ICH), 1996.

For peer review only

For peer review only - http://bmjopen.bmj.com/site/about/guidelines.xhtml

APPENDICES

Figure 1: OPTICA trial flow chart

*targeted number. In dark grey: steps done by general practitioners. In white: steps done by study team at BIHAM. Abbreviations: BIHAM, Institute of Primary Health Care of the University of Bern, GPs, general practitioners; STRIPA, Systematic Tool to Reduce Inappropriate Prescribing Assistant

For peer review only

Figure 2: OPTICA data flow chart

Step-by-step explanation of the data flow during the OPTICA trial. Abbreviations: EMR, electronic medical record; FIRE, Family medicine ICPC Research using Electronic medical records; GPs, general practitioners; OPTICA, Optimizing PharmacoTherapy In the Multimorbid Elderly in Primary CAre; STRIPA, Systematic Tool to Reduce Inappropriate Prescribing Assistant

For peer review only

Figure 1: OPTICA trial flow chart

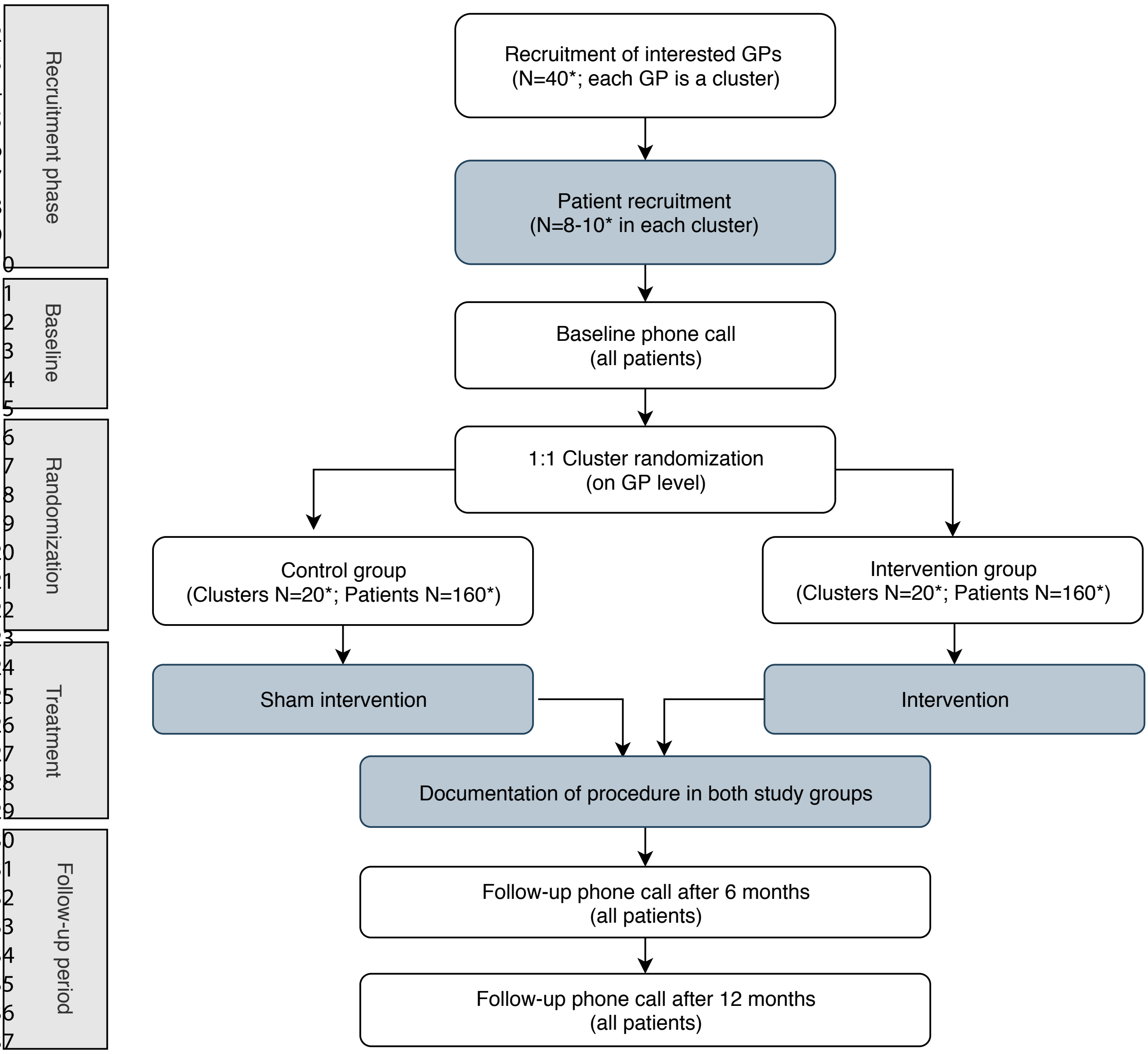

Figure 2: OPTICA data flow chart

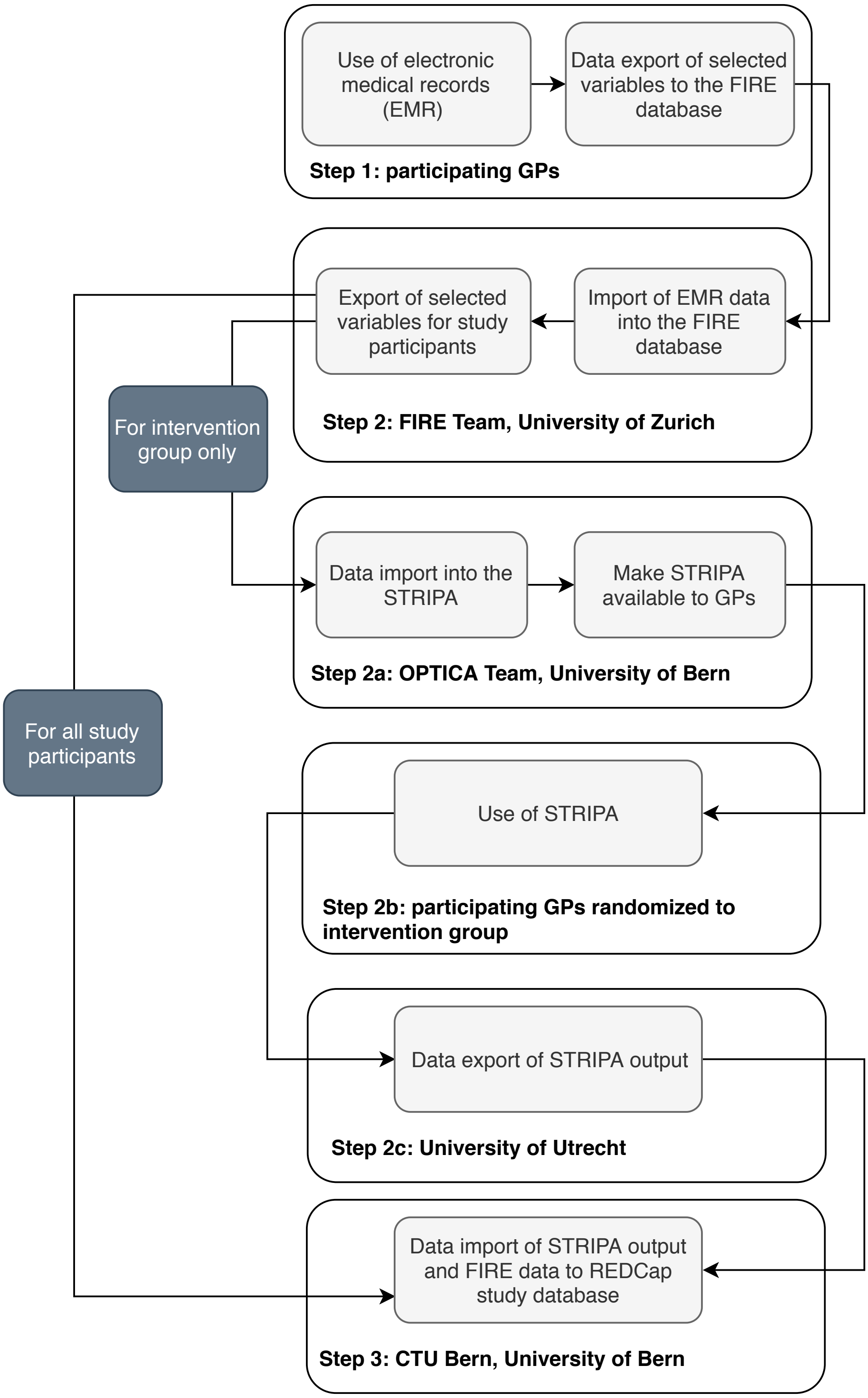

Reporting checklist for protocol of a clinical trial.

Based on the SPIRIT guidelines.

Instructions to authors

Complete this checklist by entering the page numbers from your manuscript where readers will find each of the items listed below.

Your article may not currently address all the items on the checklist. Please modify your text to include the missing information. If you are certain that an item does not apply, please write "n/a" and provide a short explanation.

Upload your completed checklist as an extra file when you submit to a journal.

In your methods section, say that you used the SPIRIT reporting guidelines, and cite them as:

Chan A-W, Tetzlaff JM, Altman DG, Laupacis A, Gøtzsche PC, Krleža-Jerić K, Hróbjartsson A, Mann H, Dickersin K, Berlin J, Doré C, Parulekar W, Summerskill W, Groves T, Schulz K, Sox H, Rockhold FW, Rennie D, Moher D. SPIRIT 2013 Statement: Defining standard protocol items for clinical trials. Ann Intern Med. 2013;158(3):200-207

		Reporting Item	Page Number
Title	#1	Descriptive title identifying the study design, population, interventions, and, if applicable, trial acronym	p.1
Trial registration	#2a	Trial identifier and registry name. If not yet registered, name of intended registry	p.2
Trial registration: data set	#2b	All items from the World Health Organization Trial Registration Data Set	p.2
Protocol version	#3	Date and version identifier	p.2
Funding	#4	Sources and types of financial, material, and other support	p.2,p.17
Roles and responsibilities: contributorship	#5a	Names, affiliations, and roles of protocol contributors	p.2, p.15
Roles and	#5b	Name and contact information for the trial sponsor	p. 1 (see note

For peer review only

responsibilities: sponsor contact information			1)
Roles and responsibilities: sponsor and funder	#5c	Role of study sponsor and funders, if any, in study design; collection, management, analysis, and interpretation of data; writing of the report; and the decision to submit the report for publication, including whether they will have ultimate authority over any of these activities	p.17 (see note 2)
Roles and responsibilities: committees	#5d	Composition, roles, and responsibilities of the coordinating centre, steering committee, endpoint adjudication committee, data management team, and other individuals or groups overseeing the trial, if applicable (see Item 21a for data monitoring committee)	p. 12
Background and rationale	#6a	Description of research question and justification for undertaking the trial, including summary of relevant studies (published and unpublished) examining benefits and harms for each intervention	p. 4
Background and rationale: choice of comparators	#6b	Explanation for choice of comparators	p.6, p.7
Objectives	#7	Specific objectives or hypotheses	p.4
Trial design	#8	Description of trial design including type of trial (eg, parallel group, crossover, factorial, single group), allocation ratio, and framework (eg, superiority, equivalence, non-inferiority, exploratory)	p.4
Study setting	#9	Description of study settings (eg, community clinic, academic hospital) and list of countries where data will be collected. Reference to where list of study sites can be obtained	p.4
Eligibility criteria	#10	Inclusion and exclusion criteria for participants. If applicable, eligibility criteria for study centres and individuals who will perform the interventions (eg, surgeons, psychotherapists)	p.5

For peer review only

Interventions: description	#11a	Interventions for each group with sufficient detail to allow replication, including how and when they will be administered	p.5-7
Interventions: modifications	#11b	Criteria for discontinuing or modifying allocated interventions for a given trial participant (eg, drug dose change in response to harms, participant request, or improving / worsening disease)	p.5-7
Interventions: adherance	#11c	Strategies to improve adherence to intervention protocols, and any procedures for monitoring adherence (eg, drug tablet return; laboratory tests)	p.6-7 (see note 3)
Interventions: concomitant care	#11d	Relevant concomitant care and interventions that are permitted or prohibited during the trial	(see note 4)
Outcomes	#12	Primary, secondary, and other outcomes, including the specific measurement variable (eg, systolic blood pressure), analysis metric (eg, change from baseline, final value, time to event), method of aggregation (eg, median, proportion), and time point for each outcome. Explanation of the clinical relevance of chosen efficacy and harm outcomes is strongly recommended	p.9-11
Participant timeline	#13	Time schedule of enrolment, interventions (including any run-ins and washouts), assessments, and visits for participants. A schematic diagram is highly recommended (see Figure)	p.9 (see note 5)
Sample size	#14	Estimated number of participants needed to achieve study objectives and how it was determined, including clinical and statistical assumptions supporting any sample size calculations	p.11
Recruitment	#15	Strategies for achieving adequate participant enrolment to reach target sample size	p.11
Allocation: sequence generation	#16a	Method of generating the allocation sequence (eg, computer-generated random numbers), and list of any factors for stratification. To reduce predictability of a random sequence, details of any planned restriction (eg, blocking) should be provided in a separate document that is unavailable to those who enrol	p.5

For peer review only

		participants or assign interventions	
Allocation concealment mechanism	#16b	Mechanism of implementing the allocation sequence (eg, central telephone; sequentially numbered, opaque, sealed envelopes), describing any steps to conceal the sequence until interventions are assigned	p.5
Allocation: implementation	#16c	Who will generate the allocation sequence, who will enrol participants, and who will assign participants to interventions	p.5
Blinding (masking)	#17a	Who will be blinded after assignment to interventions (eg, trial participants, care providers, outcome assessors, data analysts), and how	p.8-9 (refer to Table 1 in manuscript)
Blinding (masking): emergency unblinding	#17b	If blinded, circumstances under which unblinding is permissible, and procedure for revealing a participant's allocated intervention during the trial	Not applicable
Data collection plan	#18a	Plans for assessment and collection of outcome, baseline, and other trial data, including any related processes to promote data quality (eg, duplicate measurements, training of assessors) and a description of study instruments (eg, questionnaires, laboratory tests) along with their reliability and validity, if known. Reference to where data collection forms can be found, if not in the protocol	p.8, p.11
Data collection plan: retention	#18b	Plans to promote participant retention and complete follow-up, including list of any outcome data to be collected for participants who discontinue or deviate from intervention protocols	p.9
Data management	#19	Plans for data entry, coding, security, and storage, including any related processes to promote data quality (eg, double data entry; range checks for data values). Reference to where details of data management procedures can be found, if not in the protocol	p.14-15
Statistics: outcomes	#20a	Statistical methods for analysing primary and secondary outcomes. Reference to where other details of the statistical analysis plan can be found, if not in	p.11-12

For peer review only

		the protocol	
Statistics: additional analyses	#20b	Methods for any additional analyses (eg, subgroup and adjusted analyses)	Not applicable.
Statistics: analysis population and missing data	#20c	Definition of analysis population relating to protocol non-adherence (eg, as randomised analysis), and any statistical methods to handle missing data (eg, multiple imputation)	p.11
Data monitoring: formal committee	#21a	Composition of data monitoring committee (DMC); summary of its role and reporting structure; statement of whether it is independent from the sponsor and competing interests; and reference to where further details about its charter can be found, if not in the protocol. Alternatively, an explanation of why a DMC is not needed	p.12
Data monitoring: interim analysis	#21b	Description of any interim analyses and stopping guidelines, including who will have access to these interim results and make the final decision to terminate the trial	p.6 (See note 6)
Harms	#22	Plans for collecting, assessing, reporting, and managing solicited and spontaneously reported adverse events and other unintended effects of trial interventions or trial conduct	p.11
Auditing	#23	Frequency and procedures for auditing trial conduct, if any, and whether the process will be independent from investigators and the sponsor	p.14
Research ethics approval	#24	Plans for seeking research ethics committee / institutional review board (REC / IRB) approval	p.2, p.14
Protocol amendments	#25	Plans for communicating important protocol modifications (eg, changes to eligibility criteria, outcomes, analyses) to relevant parties (eg, investigators, REC / IRBs, trial participants, trial registries, journals, regulators)	p.14
Consent or assent	#26a	Who will obtain informed consent or assent from potential trial participants or authorised surrogates, and how (see Item 32)	p.5, p.8

For peer review only

Consent or assent: ancillary studies	#26b	Additional consent provisions for collection and use of participant data and biological specimens in ancillary studies, if applicable	p.5
Confidentiality	#27	How personal information about potential and enrolled participants will be collected, shared, and maintained in order to protect confidentiality before, during, and after the trial	p.14
Declaration of interests	#28	Financial and other competing interests for principal investigators for the overall trial and each study site	p.15
Data access	#29	Statement of who will have access to the final trial dataset, and disclosure of contractual agreements that limit such access for investigators	p.14
Ancillary and post trial care	#30	Provisions, if any, for ancillary and post-trial care, and for compensation to those who suffer harm from trial participation	p.15
Dissemination policy: trial results	#31a	Plans for investigators and sponsor to communicate trial results to participants, healthcare professionals, the public, and other relevant groups (eg, via publication, reporting in results databases, or other data sharing arrangements), including any publication restrictions	p.15
Dissemination policy: authorship	#31b	Authorship eligibility guidelines and any intended use of professional writers	p.15
Dissemination policy: reproducible research	#31c	Plans, if any, for granting public access to the full protocol, participant-level dataset, and statistical code	p.15
Informed consent materials	#32	Model consent form and other related documentation given to participants and authorised surrogates	see note 7
Biological specimens	#33	Plans for collection, laboratory evaluation, and storage of biological specimens for genetic or molecular analysis in the current trial and for future use in ancillary studies, if applicable	Not applicable.

Author notes

For peer review only

1. Prof. Sven Streit is the Sponsor-Investigator of the OPTICA trial

2. The role of the Sponsor Investigator, Prof. Sven Streit, is described on p. 17. The funder, the Swiss National Science Foundation, does not have any role in “collection, management, analysis, and interpretation of data; writing of the report; and the decision to submit the report for publication”.

3. GPs record in the REDCap study database if and how they have performed the intervention.

4. Usual care is permitted.

5. Refer to Figure 1.

6. There will be no interim analyses.

7. Available upon request. All documents are in German.

The SPIRIT checklist is distributed under the terms of the Creative Commons Attribution License CC-BY-ND 3.0. This checklist was completed on 02. April 2019 using https://www.goodreports.org/, a tool made by the EQUATOR Network in collaboration with Penelope.ai

For peer review only

BMJ Open

‘Optimizing PharmacoTherapy In the Multimorbid Elderly in Primary Care’ (OPTICA) to improve medication appropriateness: study protocol of a cluster randomised controlled trial

Journal:	*BMJ Open*
Manuscript ID	bmjopen-2019-031080.R1
Article Type:	Protocol
Date Submitted by the Author:	26-Jul-2019
Complete List of Authors:	Jungo, Katharina; Institute of Primary Health Care (BIHAM), University of Bern,<br>Rozsnyai, Zsofia; Institute of Primary Health Care (BIHAM), University of Bern<br>Mantelli, Sophie; Institute of Primary Health Care (BIHAM), University of Bern<br>Floriani, Carmen; Institute of Primary Health Care (BIHAM), University of Bern<br>Löwe, Axel; Institute of Primary Health Care (BIHAM), University of Bern; Department of General Internal Medicine, Inselspital, Bern University Hospital, University of Bern<br>Lindemann, Fanny; Institute of Primary Health Care (BIHAM), University of Bern<br>Schwab, Nathalie; Institute of Primary Health Care (BIHAM), University of Bern; Department of General Internal Medicine, Inselspital, Bern University Hospital, University of Bern<br>Meier, Rahel; Universität Zürich, Institute of Primary Care, University of Zurich<br>Elloumi, Lamia; Department of Information and Computing Sciences, Utrecht University; Department of Communication and Cognition, Tilburg University<br>Huibers, Corlina; Department of Geriatric Medicine, University Medical Centre Utrecht<br>Sallevelt, Bastiaan; Department of Clinical Pharmacy, University Medical Centre Utrecht<br>Meulendijk, Michiel; Department of Public Health and Primary Care, Leiden University Medical Center, Leiden<br>Reeve, Emily; Quality Use of Medicines and Pharmacy Research Centre, School of Pharmacy and Medical Sciences, Division of Health Sciences, University of South Australia; Geriatric Medicine Research, Faculty of Medicine, Dalhousie University and Nova Scotia Health Authority, Halifax, NS, Canada College of Pharmacy, Faculty of Health, Dalhousie University<br>Feller, Martin; Institute of Primary Health Care (BIHAM), University of Bern; Department of General Internal Medicine, Inselspital, Bern University Hospital, University of Bern<br>Schneider, Claudio; Department of General Internal Medicine, Inselspital, Bern University Hospital, University of Bern<br>Bhend, Heinz; Hausarzt Praxis Städtli Aarburg

	Bürki, Pius; Berufsverband der Haus- und Kinderärztinnen Schweiz<br>Trelle, S; University of Bern, CTU Bern, University of Bern<br>Spruit, Marco; Department of Information and Computing Sciences, Utrecht University<br>Schwenkglenks, Matthias; Institute of Pharmaceutical Medicine (ECPM), University of Basel; Epidemiology, Biostatistics and Prevention Institute, University of Zurich<br>Rodondi, Nicolas; Institute of Primary Health Care (BIHAM), University of Bern; Department of General Internal Medicine, Inselspital, Bern University Hospital, University of Bern<br>Streit, Sven; Institute of Primary Health Care (BIHAM), University of Bern
**Primary Subject Heading**:	General practice / Family practice
Secondary Subject Heading:	Health services research
Keywords:	Multimorbidity, Polypharmacy, PRIMARY CARE

SCHOLARONE™
Manuscripts

'Optimizing PharmacoTherapy In the Multimorbid Elderly in Primary Care' (OPTICA) to improve medication appropriateness: study protocol of a cluster randomised controlled trial

Jungo KT[1], Rozsnyai Z[1], Mantelli S[1], Floriani C[1], Löwe AL[1,2], Lindemann F[1], Schwab N[1,2], Meier R[3], Elloumi L[4,5], Huibers CJA[6], Sallevelt BTGM[7], Meulendijk MC[8], Reeve E[9,10], Feller M[1,2], Schneider C[2], Bhend H[11], Bürki P[12], Trelle S[13], Spruit M[4], Schwenkglenks M[14,15], Rodondi N[1,2], Streit S[1]

Affiliations:

[1] Institute of Primary Health Care (BIHAM), University of Bern, Bern, Switzerland

[2] Department of General Internal Medicine, Inselspital, Bern University Hospital, University of Bern, Bern, Switzerland

[3] Institute of Primary Care, University of Zurich, Zurich, Switzerland

[4] Department of Information and Computing Sciences, Utrecht University, Utrecht, The Netherlands

[5] Department of Communication and Cognition,Tilburg University,Tilburg,The Netherlands

[6] Department of Geriatric Medicine, University Medical Centre Utrecht, Utrecht, The Netherlands

[7] Department of Clinical Pharmacy, University Medical Centre Utrecht, Utrecht, The Netherlands

[8] Department of Public Health and Primary Care, Leiden University Medical Center, Leiden, The Netherlands

[9] Quality Use of Medicines and Pharmacy Research Centre, School of Pharmacy and Medical Sciences, Division of Health Sciences, University of South Australia, Adelaide, South Australia, Australia

[10] Geriatric Medicine Research, Faculty of Medicine, Dalhousie University and Nova Scotia Health Authority, Halifax, NS, Canada College of Pharmacy, Faculty of Health, Dalhousie University, NS, Canada

[11] Hausarzt Praxis Städtli Aarburg, Aargau, Switzerland

[12] Berufsverband der Haus- und Kinderärztinnen Schweiz, Bern, Switzerland

[13] CTU Bern, University of Bern, Bern, Switzerland

[14] Institute of Pharmaceutical Medicine (ECPM), University of Basel, Basel, Switzerland

[15] Epidemiology, Biostatistics and Prevention Institute, University of Zurich, Zurich, Switzerland

Corresponding author:
Professor Sven Streit, MD MSc PhD
Sponsor-Investigator
Institute of Primary Health Care (BIHAM), University of Bern, Switzerland
Mittelstrasse 43
3012 Bern, Switzerland
Phone number: +41 31 631 58 75
Email: sven.streit@biham.unibe.ch

Word count: 5762
Key words: Primary Care, Multimorbidity, Polypharmacy

For peer review only

ABSTRACT

Introduction:

Multimorbidity and polypharmacy are major risk factors for potentially inappropriate prescribing (e.g. over- and underprescribing), and systematic medication reviews are complex and time consuming. In this trial, the investigators aim to determine if a systematic software-based medication review improves medication appropriateness more than standard care in older, multimorbid patients with polypharmacy.

Methods and analysis:

OPTICA (Optimizing PharmacoTherapy In the Multimorbid Elderly in Primary Care) is a cluster randomised, controlled trial that will include outpatients from the Swiss primary care setting, aged ≥65 years with ≥3 chronic medical conditions and concurrent use of ≥5 chronic medications. Patients treated by the same general practitioner constitute a cluster, and clusters are randomised 1:1 to either a standard care sham intervention, in which the general practitioner discusses with the patient if the medication list is complete, or a systematic medication review intervention based on the use of the 'Systematic Tool to Reduce Inappropriate Prescribing'-Assistant (STRIPA). STRIPA is a web-based clinical decision support system that helps customize medication reviews. It is based on the validated 'Screening Tool of Older Person's Prescriptions' (STOPP) and 'Screening Tool to Alert doctors to Right Treatment' (START) criteria to detect potentially inappropriate prescribing. The trial's follow-up period is twelve months. Outcomes will be assessed at baseline, six, and twelve months. The primary endpoint is medication appropriateness, as measured jointly by the change in the Medication Appropriateness Index (MAI) and Assessment of Underutilization (AOU). Secondary endpoints include the degree of polypharmacy, overprescribing and underprescribing, the number of falls and fractures, quality of life, the amount of formal and informal care received by patients, survival, patients' quality adjusted life years, patients' medical costs, cost-effectiveness of the intervention, percentage of recommendations accepted by general practitioners percentage of recommendation rejected by general practitioners, and patients' willingness to have medications deprescribed.

Ethics and dissemination:

The ethics committee of the canton of Bern in Switzerland approved the trial protocol. The results of this trial will be published in a peer-reviewed journal.

Main funding:

Swiss National Science Foundation, National Research Programme (NRP 74) "Smarter Healthcare"

Trial registration numbers:

Clinicaltrials.gov: NCT03724539

KOFAM (Swiss national portal): SNCTP000003060

Universal Trial Number: U1111-1226-8013

Protocol version: v1.5, 05.05.2018

For peer review only - http://bmjopen.bmj.com/site/about/guidelines.xhtml

STRENGTHS AND LIMITATIONS

- The OPTICA trial is the first randomized controlled trial to examine the effect of the 'Systematic Tool to Reduce Inappropriate Prescribing'-Assistant (STRIPA), a software-assisted clinical decision support tool, on medication appropriateness in older, multimorbid patients with polypharmacy in a primary care setting.
- OPTICA is the first randomized controlled trial to test the use of software-based structured medication review in Swiss primary care.
- The OPTICA trial demonstrates how linked and coded data from electronic medical records can be used to evaluate primary care interventions in a randomized controlled trial setting. The investigators limit selection bias by using screening lists with a random sample of potentially eligible patients and randomising general practitioners after patient recruitment is complete, but cannot eliminate the risk of selection bias.
- The investigators will conduct a sham intervention in accordance with usual care in the control group to improve patient blinding but, by design, they could not blind general practitioners.

INTRODUCTION

Globally, there is a high prevalence of multimorbidity and polypharmacy in people more than 65 years old.[1,2] Multimorbidity is commonly defined as the co-existence of three or more chronic diseases,[3] and polypharmacy is defined as the regular intake of five or more medications.[4] Polypharmacy is often caused by multimorbidity and is linked to a high risk of potentially inappropriate prescribing,[5,6] which has three main elements, namely: 1) overuse, 2) underuse, and 3) inappropriate use (i.e. wrong dose/medication for the indication).[7]

Appropriate polypharmacy denotes a situation in which “medication use is optimised according to the patients’ clinical needs” and in which patients “receive the most appropriate combinations of medications based on the best available evidence”.[8] While appropriate polypharmacy can improve quality of life and prevent consequences of disease, inappropriate polypharmacy can harm patients’ health.[4] For instance, it can increase the risk of falls and fractures,[9] lead to cognitive decline,[10,11] and it can reduce quality of life.[9] Polypharmacy also increases the risk of drug-drug interactions, drug-disease interactions, and adverse drug events.[12-15] Treatment of older patients with multimorbidity and polypharmacy is a complex problem in primary care and other medical fields and both conditions are increasingly common as populations age. Due to the relatively small number of randomized controlled trials on different interventions for the management of multimorbid people, there currently remain uncertainties about the effectiveness of these interventions.[16] Medication reviews in older adults with multimorbidity and polypharmacy can be complicated and time-consuming.[17] General practitioners (GPs) have reported that time

For peer review only - http://bmjopen.bmj.com/site/about/guidelines.xhtml

limitations and lack of user-friendly and reliable tools pose significant barriers to regular medication optimization activities in practice.[18]

Current evidence is ambiguous on whether review interventions that aim at optimizing medication can reduce inappropriate polypharmacy. A systematic review and meta-analysis of short-term medication review interventions published in 2017 showed that such isolated medication reviews have an impact on drug-related outcomes, but only minimally influence clinical outcomes and have no impact on quality of life.[19] A more recent systematic review and meta-analysis on interventions to improve the appropriateness of medication use in older people showed that such interventions may be beneficial for reducing potential prescribing omissions, but that it remains uncertain whether they improve the appropriateness of medication use.[20]

The increasing use of electronic medical records (EMR) and electronic prescribing has opened opportunities to incorporate web-based clinical decision support systems (CDSS) at the point of care. For instance, a complex intervention with an informatics tool and financial incentives has led to a reduction in high-risk prescribing of nonsteroidal anti-inflammatory drugs and antiplatelet medications.[21] Medication optimization studies that use clinical decision support systems enabled by electronic medical records have shown that the interventions are feasible and acceptable to clinicians, but evidence that they lead to more appropriate use of medications in general and improve clinical outcomes is still limited.[22]

Objectives

The primary aim of the cluster-randomised 'Optimizing PharmacoTherapy In the Multimorbid Elderly in Primary Care' (OPTICA) trial is to test whether the use of a systematic, software-assisted medication review intervention leads to a more appropriate use of medications than a usual care sham intervention. This primary outcome will be measured by the Medication Appropriateness Index (MAI) and the Assessment of Underutilization (AOU). Secondary outcomes are the degree of polypharmacy, the degree of overprescribing, the degree of underprescribing, the number of falls and fractures, quality of life, the amount of formal and informal care received by patients, survival, patients' quality adjusted life years, patients' medical costs, cost-effectiveness of the intervention, percentage of recommendations accepted and rejected by GPs, and patients' willingness to have medications deprescribed.

METHODS AND ANALYSIS

General study design and setting

The OPTICA trial is a cluster randomised controlled trial (RCT), coordinated at the Institute of Primary Health Care of the University of Bern (BIHAM). Participating GPs, who will each systematically recruit multimorbid, older patients with polypharmacy, define the clusters. Through

For peer review only - http://bmjopen.bmj.com/site/about/guidelines.xhtml

randomisation, the GPs will be allocated to the structured medication review or a usual care based sham intervention.

The investigators used the SPIRIT checklist when they wrote this protocol.[23] Please refer to Figure 1 for the study flow chart and Figure 2 for a description of data flow within the OPTICA trial.

Cluster definition

The trial will be conducted in about 40 primary care offices in the German-speaking regions of Switzerland. Each participating GP, who prescribes the medication of his/her patients, constitutes a cluster. GPs were recruited as sub-investigators from October 2017 until June 2018 from the pool of teaching physicians at the University of Bern and from the group of GPs who attended project presentations hosted by the BIHAM throughout 2017 and 2018. Additional GPs will be recruited throughout 2019 to replace GPs who had to withdraw from the study, as the data export to the FIRE database did not work in their GP office. Engagement with GPs started early in anticipation of slow recruitment, which has been reported in previous studies.[24,25] All eligible GPs who showed interest in the OPTICA trial were visited by the investigators at their GP office and given a detailed explanation of the trial.[26] As of July 2019, 83% of the participating GPs are male. Eighty percent of the GPs work in group practice, while 20% work in single practice. Twenty-nine percent of GPs work in the countryside and 71% work in urban and sub-urban areas.

Randomisation

Randomisation is done after the cluster has been completed. The randomisation is done centrally in a web-based system (REDCap) by a study team member after all cluster information has been entered. Each participating GP is allocated 1:1 to the intervention group or the control group, using unstratified randomisation with a random sequence of block sizes of two and four. An independent statistician, who is otherwise not involved in the trial, generated the randomisation list. To uphold the concealment of allocation only system administrators who are otherwise not involved in the trial can access the randomisation list.

Eligibility criteria

Inclusion criteria

General practitioner level: To be eligible for participation, GPs must be participating in the “Family medicine ICPC Research using Electronic medical records” (FIRE) project led by the Institute of Primary Care of the University of Zurich (IHAMZ).[27] The electronic medical records (EMR) they use in their GP office have to be compatible with the FIRE database, which is a database with anonymous data from the EMR of more than 500 GPs in Switzerland. The database of the FIRE project contains administrative data, vital data, laboratory values, International Classification of

For peer review only - http://bmjopen.bmj.com/site/about/guidelines.xhtml

Primary Care 2 (ICPC-2) codes for diagnoses and information on medications prescribed. Participating GPs can, only for the purpose of this trial, identify individual patients in the usually anonymous FIRE database, so that their data can be used in this trial. All participating GPs must complete an online training for Good Clinical Practice (GCP).

Patient level: Patients must be enlisted by one of the participating GPs, so that this GP is their main prescribing physician and they must see their GP regularly. The investigators left the definition of regularity to the judgment of participating physicians. Patients must be ≥65 years of age, have ≥3 chronic diseases based on ICPC-2 coding or based on the GP's clinical decision (multimorbidity), and regularly take ≥5 medications (polypharmacy). Written informed consent must be sought from patients or, if patients suffer from cognitive impairment, their relative prior to enrolment. This consent includes the right to obtain data from the FIRE project about the study participant.

Exclusion criteria

General practitioners level: In group practices, only one GP can take part in the trial.

Patient level: To maximize the generalizability of the study population, the investigators kept exclusion criteria to a minimum. They excluded patients currently taking part in another interventional study.

Intervention

The intervention, a systematic medication review, includes the use of a web-based clinical decision support system called 'Systematic Tool to Reduce Inappropriate Prescribing'- Assistant (STRIPA), developed by a team from the Utrecht University and the University Medical Center Utrecht in the Netherlands.[28] STRIPA is based on all algorithms of the 'Screening Tool of Older Person's Prescriptions' (STOPP) and 'Screening Tool to Alert doctors to Right Treatment' (START) criteria (version 2),[29] which are expert-consensus lists of inappropriate and appropriate medications for older adults that consider co-existing medical conditions and are thus suited for optimizing the prescriptions of multimorbid, older patients.[30] STRIPA combines implicit and explicit prescribing tools and takes a stepped approach that actively encourages patient involvement in decision-making.[28,31] Taking into account medications, diagnoses, laboratory values, and vital data, the STRIPA generates recommendations for physicians about "underprescribing, ineffective prescribing, overprescribing, side effects, contraindications, (...) drug-drug and drug-disease interactions, incorrect dosages/dosing frequencies, and practical intake issues".[28] The intervention's recommendations allow patients and GPs to conduct a shared decision-making process about patients' medication intake, so patient preferences play an important role in this trial.

For peer review only - http://bmjopen.bmj.com/site/about/guidelines.xhtml

A STRIPA validation study with GPs based on two test cases showed appropriate prescribing decisions increased and inappropriate decisions decreased during a medication review of an older, multimorbid patient with polypharmacy.[31] STRIPA is being tested in the European multicentre clinical trial 'OPtimising thERapy to prevent Avoidable hospital admissions in Multimorbid older people' (the OPERAM trial) in the hospital setting to find out if it can reduce drug-related hospital re-admissions.[32]

This study adapted the intervention from the OPERAM trial so it could be tested in the primary care setting.[33] The intervention comprises six steps:

1. This data will be imported from the FIRE database into the STRIPA: patients' baseline characteristics (e.g., sex, age); vital data and laboratory measurements (e.g., kidney function, blood pressure), patients' signs and symptoms, different scores (e.g., HASBLED), ICPC-2 coded diagnoses, and medications entered as Anatomical Therapeutic Chemical (ATC) codes.
2. GPs log in to the 'Systematic Tool to Reduce Inappropriate Prescribing'- Assistant (STRIPA) and see a list with their recruited patients' identifiers. When they click on a patient's ID, the information exported from the EMR to STRIPA via FIRE becomes visible (from Step 1). The GP can add unrecorded values or adjust values (e.g., when more recent laboratory values are available). This might be necessary because it might take some time to obtain the FIRE data and to import it to STRIPA and hence the information might change in the meantime.
3. GPs use a drag and drop function to link each medication to a diagnosis.
4. GPs run the analysis in STRIPA and then look at STRIPA's recommendations. After the analysis is finished, STRIPA records the recommendations it generates so that they will be available for later analysis and generates a PDF report that can be saved by GPs.
5. GPs then must decide if they agree with STRIPA's advice given and if they will present it to their patient. They can follow, partly follow, or decline STRIPA's recommendations.
6. At the next appointment with the same patient, GPs will present the recommendations that they consider appropriate. GP and patient will decide together, in a shared decision-making process, which recommendations to implement. GPs will work from a checklist that explains how to conduct the shared decision-making process so that the patients' preferences are met. The shared-decision making process is based on Elwyn et al.'s model[34] and was adapted from the OPERAM trial. The four key elements of this shared decision-making process are "choice talk", "option talk", "preference talk", and "decision talk".[34] Patients may follow, partly follow or decline the recommendations. Because multimorbid patients are likely to have multiple prescribers, GPs and patients can discuss recommendations with other prescribers, e.g., specialists. The GP records in the REDCap

For peer review only - http://bmjopen.bmj.com/site/about/guidelines.xhtml

study database the patient's decisions and the reasons recommendations were accepted, partially accepted, or declined. These records will be analysed later.

Before the intervention, GPs in the intervention group will watch an instruction video and read training material that will guide them through the intervention step-by-step.

Sham intervention

Patients assigned to the control arm receive a sham intervention from their GP - a medication discussion in accordance with usual care. This means that the GP will, as usual in standard care, ask the patient if the current list of medication is complete or needs to be adapted, then share decision making with the patient about possible changes. The GPs who do the sham intervention will receive the same shared decision-making checklist as the GPs in the intervention group. The investigators explicitly ask GPs not to deviate from their usual practice when they review the patients' medication and not to use additional tools. After the sham intervention, GPs record procedures, decisions, and reasons in the REDCap study database for later analysis. This sham intervention ensures that the medication list of control group study participants, which will be used to assess study outcomes, is up-to-date, while also ensuring patients are blinded.

Screening and enrolment

For patient recruitment, the investigators use data from the FIRE project to prepare a screening list for each GP that contains a random selection of eligible patients. On each screening list, there are about 20 patients who were randomly sampled from all enlisted patients after they met the age criteria and were determined to regularly take at least five chronic medications, based on the Pharmacy-based Cost Group (PCG) model.[35-37] Since ICPC-2 coding is suboptimal in many GP offices, the investigators decided not to include the chronic disease criterion when they prepare the screening lists. From the list, GPs systematically recruit eight to ten patients who meet the full inclusion criteria, which now include the number of chronic diseases, and do not meet any of the exclusion criteria. GPs will then recruit patients starting either at the bottom of the list or the top. If GPs have finished the first screening list without recruiting the minimum amount of participants, the investigators will provide them with a second list.

Since the screening lists are compiled from a random patient sample from each GP office based on FIRE data, patients on the list may have been treated by a different GP who works in the same group practice. It is also possible that patients on the list changed their GP or have died. Since prescriptions frequently change[38], some patients with polypharmacy might not have qualified for the screening list when data was last exported. For all these reasons, the protocol allows GPs to skip patients on the screening list if they provide an explanation, or to recruit patients who are not on the list but fulfil the inclusion and do not meet the exclusion criteria. After identifying patients on the list and verifying their eligibility, GPs then inform the patient about the study and seek their

For peer review only - http://bmjopen.bmj.com/site/about/guidelines.xhtml

informed consent. Study-related appointments are added to the patients' regular appointments at no charge.

Data collection

Study participants or their relatives are followed up by phone at baseline and at six and twelve months after study enrolment to complete data collection. At these times, complementary information from the FIRE database, including information about medications and chronic diseases, is imported into the REDCap study database.[27]

Blinding procedures

The OPTICA trial is blinded to the extent the cluster design of this randomised controlled trial allows. Our method of partial blinding, which is similar to that used in the OPERAM trial,[32] is set out in detail in Table 1.

Table 1: Blinding status and measures to assure blinding

Role	Blinding status	How to achieve blinding
General practitioners	Unblinded	When screening for patients and seeking informed consent, GPs are still blinded, as each cluster (GP) will be only randomised after the cluster is full (8-10 patients). The screening list GPs use for recruitment contains a random sample of potentially eligible patients, which was generated from FIRE data by a blinded study team member. All this is done to prevent selection bias. However, since GPs can recruit patients outside this list, not all study participants are recruited randomly.<br>However, our study design makes it impossible to blind GPs throughout the trial. When GPs do the medication review/discussion, they know what group they are allocated to. Nevertheless, GPs in the control arm do not know the procedures in the intervention arm, which prevents cross-contamination; if they knew the procedure in the intervention arm, they might adapt their usual care.
Data collectors and assessors	Blinded	The randomisation of GPs is kept concealed from the team that makes follow-up calls to avoid interviewer bias. Data collectors and assessors have no access to unblinded study information in the database or to local source data. If a serious adverse event (SAE) occurs, the study coordinator and project manager will be informed.
Data manager and data analysist	Unblinded	The investigators cannot blind our data managers and analysts because they can see the differences in data structure between the study groups. This is why the investigators use a new data analyst to prepare a clean data set with truncated data to conduct a blinded analysis of the primary outcome.

For peer review only - http://bmjopen.bmj.com/site/about/guidelines.xhtml

Study coordinator and project manager, including principal investigator	Unblinded	The study coordinator and project manager of the trial know the treatment allocation. They are responsible for collecting information about SAEs and performing safety assessments.
Patients	Partially blinded	Patients stay partially blinded. They are only given a “high-level description” of the study question so they know that their GP has been allocated to one of two study groups, but they do not know which one. To uphold patient blinding, patients in the control group will meet their GP for a medication discussion and a shared decision-making about their prescriptions. This means patients in each study arm see GPs the same number of times during the trial and cannot guess their allocation status based on the number of consultations.
The OPTICA trial’s approach to blinding resembles the approach used in the OPERAM trial.[32]; Accronyms: GP, General Practitioner; SAE, serious adverse event		

Follow-up

Outcome information is collected at baseline, at six, and at twelve months via telephone calls with patients or relatives conducted by a blinded study team member, and through FIRE database exports. The study team will make every reasonable effort to keep each patient in the study until all planned treatments and assessments have been performed. But patients may withdraw from the study or be withdrawn when they are lost to follow-up.

Assessment of primary outcome

The primary endpoint is medication appropriateness, measured jointly by the Medication Appropriateness Index (MAI) and the Assessment of Underutilization (AOU), in each study group. While the AOU assesses underprescribing, the MAI is a tool to assess different elements of medication prescribing (e.g. overprescribing, drug-drug interactions, etc.).[7,39,40]

Blinded study teammembers assess the MAI for each regular medication study participants take and assess the AOU for each chronic condition study participants have. The investigators will use the weighted 10-item version of the MAI developed by Samsa et al.[40] However, due to the rapidly changing drug prices the MAI item on cost-effectiveness will not be included. The criteria of the MAI, including corresponding weights, can be found in table 2. Using clinical data and the predefined operational definitions for each item, the assessor rates each medication on a three-point scale ranging from A = appropriate, B = marginally appropriate, C = inappropriate. Each “inappropriate” rating will receive the respective weight from Table 2, while the weight of ratings “appropriate” and “marginally appropriate” will be 0. Thus, the score for each medication ranges from 0 to 17 (as the cost-effectiveness criterion will not be included).[40] A higher score indicates a greater degree of medication inappropriateness. The investigators will calculate the score for each medication and then calculate the summated score for each patient, by summing up the

For peer review only - http://bmjopen.bmj.com/site/about/guidelines.xhtml
For peer review only

scores for each medication. For the AOU, assessors decide for each chronic condition if there is i) no omission, ii) marginal omission, or iii) omission of indicated medication.[41-43] For each patient, the investigators will calculate how many omissions there are.

Table 2: Criteria of the Medication Appropriateness Index including weights

	Item	Weight
1	Is there an indication for the drug?	3
2	Is the medication effective for the condition?	3
3	Is the dosage correct?	2
4	Are the directions correct?	2
5	Are the directions practical?	1
6	Are there clinically significant drug-drug interactions?	2
7	Are there clinically significant drug-disease/condition interactions?	2
8	Is there unnecessary duplication with other drug(s)?	1
9	Is the duration of therapy acceptable?	1

Reference: Samsa GP, Hanlon JT, Schmader KE, et al. A summated score for the medication appropriateness index: development and assessment of clinimetric properties including content validity. Journal of clinical epidemiology 1994;47(8):891-6. / Item 10 "Is this drug the least expensive alternative compared to others of equal utility?" has been excluded.

The MAI and the AOU will be assessed and calculated for baseline, the follow-up 1 at 6 months and the follow-up 2 at 12 months. For at least 32 cases (10% of the targeted sample size), two blinded investigators will conduct a blinded independent double assessment of the MAI and the AOU to check inter-rater reliability. The investigators will import information about diagnoses (coded in ICPC-2) and medication intake from the FIRE database into the study database and use it for these assessments.

Assessment of secondary outcomes

The following secondary outcomes are assessed at baseline, six, and twelve months (data source in brackets):

- Degree of polypharmacy; i.e., the number of regular long-term medications patients take (FIRE database)
- Degree of overprescribing, measured by the Medication Appropriateness Index (MAI) (assessment of FIRE data done by study team)
- Degree of underprescribing, measured by the Assessment of Underutilization (AOU) (assessment of FIRE data done by study team)
- Number of falls and fractures in the last six months (patient/relative phone call)
- Quality of life, based on the 5-level version of the European Quality of Life-5 Dimensions questionnaire (EQ-5D), including pain/discomfort[44,45] (patient/relative phone call)
- Amount of formal care received: number and length of stay of planned and unplanned hospitalizations; visits to the emergency room without inpatient hospitalization; GP visits; medical specialist visits (differentiated by specialty); hospital outpatient visits; inpatient stays; length of stay at rehabilitation facilities; physiotherapist and other allied therapist

For peer review only

visits; nursing home admissions (in patients who were living in the community at baseline); length of stay in nursing homes; and, number of home nursing visits (patient/relative phone call, except for number of GP visits which comes from FIRE database)

- Amount of informal care received: unpaid care by, e.g., family members, relatives, friends (patient/relative phone call)
- Survival (patient/relative phone call or reported by GP)
- Quality-adjusted life years (QALYs) accrued in one year,[46] measured as a function of the length and the quality of life (calculated based on data from patient/relative phone call)
- Direct medical costs accrued in one year, measured by combining formal and informal healthcare resource use observed in the trial (patient/relative phone call), including the time GPs spend on the intervention (reported by GP) and software costs (literature search), and Swiss unit costs from sources external to the trial (literature search)
- Cost-effectiveness of the intervention, calculated by combining clinical data (FIRE database), quality of life data (patient/relative phone call), and healthcare use data collected in the trial (patient/relative phone call)

The following outcome will be assessed after the intervention:

- Percentage of STRIPA recommendations accepted and rejected by GPs (reported by GPs, cross-verified with STRIPA reports)

The following secondary outcome is assessed at baseline only:

- Patients' willingness to have medications deprescribed, measured with the validated 'revised Patient Attitudes Towards Deprescribing' (rPATD) questionnaire[47,48] (patient/relative phone call)

Safety outcomes include adverse events, serious adverse events and device deficiencies.

Study duration

GPs can recruit the patients over a period of at least six months, so they can integrate recruitment into their daily practice. Recruited patients are followed up for one year.

Sample size

Sample size calculation is based on testing the two co-primary outcomes for superiority and uses the Bonferroni-approach to account for multiple testing. Based on trial results published by Gallagher et al. in 2011, the investigators assumed that 35% of patients in the control group and 60% of patients in the intervention group will improve their total Medication Appropriateness Index (MAI) score (at least one less point) and that 10% of patients in the control group and 30% of patients in the intervention group will have a better Assessment of Underutilization (AOU) score (at least one fewer prescribing omission).[49] Intra-cluster correlations (ICC) of 0.01 to 0.05 are

For peer review only - http://bmjopen.bmj.com/site/about/guidelines.xhtml

typically found for binary outcomes in elderly individuals.[50] The investigators conservatively assumed an ICC of 0.05 to calculate sample size. The investigators fixed the type I error at a Bonferroni-corrected two-sided alpha level of 0.025.

Based on a two-sample comparison of proportions and a pre-specified number of clusters of about 40 (about 20 per arm), seven patients per cluster are required to detect a difference of 25% between the two groups in the proportion of improvement in the MAI, with a power of 90%. The number of 40 GPs was selected arbitrarily for feasibility reasons. Using the same assumption for the AOU, the investigators found they also need seven patients per cluster to detect a difference of 20%. The investigators thus need a sample of 280 patients (140 per arm) to provide 81% power to detect a significant improvement in both the MAI score and the AOU. To account for attrition from dropout or death (15% estimated), the number of patients per cluster was increased to 8 (max. 10), so the final sample size should be about 320 patients (160 per arm). The investigators will closely support GPs to help them reach our target sample size.

Statistical analysis

In case data on outcomes are incomplete, the investigators will use multiple imputation to replace missing values, taking data clustering into account. No interim analyses are planned.

There are two co-primary outcomes: improvement in the MAI at twelve months, defined as decrease of at least one point, and improvement in the AOU at twelve months, defined as at least one less prescribing omission. Both outcomes will be tested separately; success is indicated by the significance of at least one of the two tests.

For the co-primary outcomes, the investigators will present and compare the proportion of patients whose MAI and AOU score improved in the control and intervention group. The relative difference between groups will be determined in a mixed-effects logistic model with a random intercept at the GP level to account for clustering. The effect measure for the primary outcomes will be odds ratios. The relative difference will be presented as odds ratio with a 95% confidence interval. The primary analysis will be an intention-to-treat (ITT) analysis. In a per-protocol (PP) analysis, the investigators will only evaluate patients who adhered to the protocol and exclude patients who violate any inclusion criteria.

Secondary binary outcomes will be evaluated like the primary outcomes. Secondary continuous outcomes will be analysed using random-effects linear regression with a random intercept at the GP level. Models will also be adjusted for the baseline value as a covariate. Secondary count outcomes will be analysed using random-effects Poisson regression with a random intercept at the GP level.

Health economic analyses will assess i) resource use and cost differences between the trial arms, ii) differences in quality-adjusted lifetime between the trial arms, expressed as quality-adjusted

For peer review only - http://bmjopen.bmj.com/site/about/guidelines.xhtml

life years [QALYs], and iii) a comparison of cost-effectiveness between intervention and standard care.

Because cluster-randomisation may not balance characteristics between groups to match individual-level randomisation, the investigators will adjust each model for patient- and physician-level variables to account for case-mix differences between groups and potential recruitment bias in a sensitivity analysis. They will also account for the correlated nature of data among GPs by using multilevel mixed-effects models. Unadjusted models will be provided for information only.

PATIENT AND PUBLIC INVOLVEMENT

Multimorbid, older patients with polypharmacy are represented in the independent Safety and Data Monitoring Board of the OPTICA trial. Throughout the trial, the Safety and Data Monitoring Board, which will consist of one GP, one statistician, and one multimorbid, older layperson with polypharmacy, will meet regularly to discuss safety and data management issues. Patients are not actively involved in recruiting study participants, but play a key role in shared decision-making during the intervention and sham intervention. The investigators have created a priority list of questionnaire components to reduce the burden of the intervention on very old and sick study participants by reducing the duration of the follow-up calls where necessary. At the end of the study, the investigators will disseminate the results to study participants in a letter.

DISCUSSION

This protocol paper highlights the features of the OPTICA trial, the first RCT in primary care to test an intervention based on the STRIPA clinical decision support system, which helps GPs customize medication reviews and optimize polypharmacy.

This clinical trial compares the effect of a structured medication review on medication appropriateness in a Swiss primary care setting and compares it to a sham intervention. Systematic medication review may facilitate shared decision-making and improve medication appropriateness, especially for GPs who treat complex multimorbid patients with polypharmacy. It may also improve patients' quality of life and health economic outcomes. This trial will add to the literature, as it examines in a real life setting a software-based intervention, which implements the STOPP/START criteria, based on data from electronic medical records. If successful, this study will demonstrate the usefulness of an electronic database, with coded data collected routinely in primary care, to be used in a clinical decision support tool. Additionally, it also focuses on multimorbid patients who are often excluded from trials.

OPTICA is subject to the following limitations:

- Despite taking precautions to avoid and reduce selection bias (cluster randomisation to avoid learning effect, screening lists for patient recruitment, randomisation of GPs after patient recruitment), selection bias is still possible because of the study design. Since

For peer review only - http://bmjopen.bmj.com/site/about/guidelines.xhtml

GPs can recruit patients outside of the screening list for practical reasons, not all patients are recruited randomly. Patients who are more engaged and/or more likely to adhere to advice may be more likely to be enrolled in the trial, which might decrease the representativeness of the study population and the generalizability of our results. The investigators will use data from FIRE to compare the characteristics of study participants with those of non-participants.

- The investigators chose a usual care sham intervention in the control group to improve patient blinding, but this design does not eliminate the risk GPs in the control group will be contaminated by the thematic of the trial (risk of deviating from their previous routine prescribing practices). This sham intervention also might lead GPs to suggest medication changes that could improve the appropriateness of patients' medications.
- Outcome assessment is based on self-reported data from patients and relatives, and on data from FIRE, so some events may be missed. To ensure FIRE data is as complete as possible, GPs must code their patient's medication intake and their diagnoses correctly. Diagnoses require ICPC-2 codes. GPs were instructed, in face-to-face meetings, about how to code and update data in their EMR for patients in this trial.[26]
- Like all interventions that take an eHealth approach (STRIPA in this case), people have different perceptions of what is user-friendly. New procedures may seem complicated in comparison to usual care. The investigators tried to simplify the process by i) using EMR data so that GPs do not have to enter all the data themselves, but only update them if needed; and ii) giving detailed instructions for using STRIPA in writing and in an online video training.
- Unrestricted randomisation designs, such as the block randomization used in this trial, are more likely to result in imbalance of factors by chance.[51]
- Another limitation of the study is the restriction to self-control during the intervention, as it is a mono-professional intervention, and that the scope of the intervention is limited to the use of the software-based clinical decision support system. However, due to the structure of the Swiss primary health care setting this design is feasible in a real life setting, whereas multi-professional interventions would be difficult to organize.
- The primary outcome of this trial is not directly patient-relevant. However, directly patient-relevant outcomes, such as quality of life, figure among the secondary outcomes of this trial.

It may be difficult to follow-up multimorbid, older patients, who are often excluded from trials[52]. The investigators believe that the task is worth the effort because trial results need to be generalizable to exactly this population. To reduce the duration of the phone calls with particularly weak study participants only the core elements of the study questionnaire will be used, if necessary, based on a predefined priority list. The OPTICA trial has the following strengths:

For peer review only - http://bmjopen.bmj.com/site/about/guidelines.xhtml

- The OPTICA trial is the first RCT to examine the effect of STRIPA on medication appropriateness in older, multimorbid patients with polypharmacy in a primary care setting.
- OPTICA is the first RCT to test the use of software-based structured medication review in Swiss primary care.
- OPTICA demonstrates the usefulness of coding and linking data from EMR, and re-using this data to evaluate primary care interventions in a randomized controlled trial setting.
- In the OPTICA trial, the investigators do not exclude patients with cognitive impairment if a relative gives their informed consent, because patients with cognitive impairment are especially prone to polypharmacy, yet they have been excluded from many other trials.

Conclusion

The OPTICA trial will compare the effect of the systematic medication review that uses STRIPA and medication discussions between patients and GPs to usual care (sham intervention) with the goal of improving medication appropriateness. The investigators expect the intervention to improve the quality of life and health status of a rapidly aging population with increasing multimorbidity and polypharmacy. The study results will inform other studies and interventions designed to optimize medication use by integrating a clinical decision support system with electronic medical records.

CURRENT STATUS OF THE OPTICA TRIAL

The OPTICA trial began recruiting in December 2018. By early July 2019, 278 patients (about 85% of the target sample size) have been recruited. The investigators have randomised 31 out of about 40 GPs. Last patient out is expected in the second half of 2020.

ETHICS

The ethics committee of the canton of Bern (Switzerland) and the Swiss regulatory authority (Swissmedic) approved the study protocol and other documentation on study conduct (BASEC ID: 2018-00914). The ethics committee and Swissmedic will receive annual safety reports and information about study stops/end and protocol amendments, as per local requirements.

All participant data will be handled according to the principles of the Declaration of Helsinki.[53] The OPTICA trial complies with all applicable standards of the ICH-GCP guideline.[54]

Data management, monitoring, safety reporting and audits meet the requirements of Swiss law. The investigators uphold the principle of patients' right to privacy and comply with applicable privacy laws. Confidentiality and anonymity of the patients shall be guaranteed when the data is

For peer review only

For peer review only - http://bmjopen.bmj.com/site/about/guidelines.xhtml

presented at scientific meetings or published in scientific journals. Only selected study team members will have access to the final trial dataset.

Risks, including human failure and software malfunction, cannot be excluded. But STRIPA only makes prescription recommendations to GPs in the intervention group. Participating GPs are experienced (mean age of experience as GP: 16 years), and will take the final decision about whether to accept the recommendations and to present them to the patient. Patients thus are not exposed to more risk than they would be in standard care. This clinical trial should entail minimal risk for participants and the benefit-risk ratio is positive.

Basler Versicherungen will provide insurance and cover eventual damages.

Participating GPs have signed a non-disclosure agreement.

DISSEMINATION

OPTICA embraces an open access policy and will vigorously disseminate all resulting data, study results, and publications. The investigators closely collaborate with National Research Programme 74 (NRP74) to optimise dissemination of the study results to the public.

AUTHOR CONTRIBUTIONS

Authorship eligibility is based on the four ICMJE authorship criteria.

Study concept and design: Trelle, Spruit, Schwenkglenks, Jungo, Bhend, Meier, Rodondi, Streit

Drafting of the manuscript: Jungo, Streit

Critical contribution to the study protocol and revision of the manuscript for important intellectual content: Mantelli, Floriani, Löwe, Lindemann, Rozsnyai, Schwab, Meier, Sallevelt, Huibers, Meulendijk, Elloumi, Schneider, Feller, Bhend, Reeve, Bürki, Trelle, Spruit, Schwenkglenks, Rodondi

Statistical analysis: Trelle

Obtained funding: Rodondi, Streit

Administrative, technical, or material support: Schwab

Supervision: Streit

FUNDING STATEMENT

This work is supported by the Swiss National Science Foundation, within the framework of the National Research Programme 74 (NRP74) under contract number 407440_167465 (to Prof. Sven Streit and Prof. Nicolas Rodondi).

DATA SHARING STATEMENT

For peer review only - http://bmjopen.bmj.com/site/about/guidelines.xhtml

Data will be deposited in the BORIS data repository and shared upon request to the Sponsor-Investigator only. No data are currently available.

COMPETING INTERESTS STATEMENT

The authors state that they have no competing interest with regard to the intellectual concern and proprietary affairs.

CONVEYANCE AND PEER REVIEW

The trial protocol has been peer-reviewed for ethical, regulatory, and funding approval before submission.

ACKNOWLEDGEMENT

The entire OPTICA study team would like to acknowledge the enormous help provided by all general practitioners participating in OPTICA and for their efforts to recruit older multimorbid patients with polypharmacy. They also thank Kali Tal for her editorial suggestions.

For peer review only

REFERENCES

1. Violan C, Foguet-Boreu Q, Flores-Mateo G, et al. Prevalence, determinants and patterns of multimorbidity in primary care: a systematic review of observational studies. *PloS one* 2014;9(7):e102149. doi: 10.1371/journal.pone.0102149 [published Online First: 2014/07/23]
2. Rawle MJ, Richards M, Davis D, et al. The prevalence and determinants of polypharmacy at age 69: a British birth cohort study. *BMC geriatrics* 2018;18(1):118. doi: 10.1186/s12877-018-0795-2 [published Online First: 2018/05/18]
3. Harrison C, Britt H, Miller G, et al. Examining different measures of multimorbidity, using a large prospective cross-sectional study in Australian general practice. *BMJ open* 2014;4(7):e004694. doi: 10.1136/bmjopen-2013-004694 [published Online First: 2014/07/13]
4. Gnjidic D, Hilmer SN, Blyth FM, et al. Polypharmacy cutoff and outcomes: five or more medicines were used to identify community-dwelling older men at risk of different adverse outcomes. *Journal of clinical epidemiology* 2012;65(9):989-95. doi: 10.1016/j.jclinepi.2012.02.018 [published Online First: 2012/06/30]
5. Mannucci PM, Nobili A. Multimorbidity and polypharmacy in the elderly: lessons from REPOSI. *Internal and emergency medicine* 2014;9(7):723-34. doi: 10.1007/s11739-014-1124-1 [published Online First: 2014/08/29]
6. Naples JG, Hajjar ER. Multimorbidity and Polypharmacy. In: Stegemann S, ed. Developing Drug Products in an Aging Society: From Concept to Prescribing. Cham: Springer International Publishing 2016:549-61.
7. Hanlon JT, Schmader KE, Ruby CM, et al. Suboptimal prescribing in older inpatients and outpatients. *J Am Geriatr Soc* 2001;49(2):200-9. [published Online First: 2001/02/24]
8. Cadogan CA, Ryan C, Hughes CM. Appropriate Polypharmacy and Medicine Safety: When Many is not Too Many. *Drug Saf* 2016;39(2):109-16. doi: 10.1007/s40264-015-0378-5 [published Online First: 2015/12/23]
9. Pan H-H, Li C-Y, Chen T-J, et al. Association of polypharmacy with fall-related fractures in older Taiwanese people: age- and gender-specific analyses. *BMJ open* 2014;4(3):e004428. doi: 10.1136/bmjopen-2013-004428
10. Niikawa H, Okamura T, Ito K, et al. Association between polypharmacy and cognitive impairment in an elderly Japanese population residing in an urban community. *Geriatrics & gerontology international* 2017;17(9):1286-93. doi: 10.1111/ggi.12862 [published Online First: 2016/09/16]
11. Oyarzun-Gonzalez XA, Taylor KC, Myers SR, et al. Cognitive Decline and Polypharmacy in an Elderly Population. *Journal of the American Geriatrics Society* 2015;63(2):397-99. doi: doi:10.1111/jgs.13283
12. Lavan AH, Gallagher P. Predicting risk of adverse drug reactions in older adults. *Therapeutic advances in drug safety* 2016;7(1):11-22. doi: 10.1177/2042098615615472 [published Online First: 2016/02/03]
13. Castilho ECD, Reis AMM, Borges TL, et al. Potential drug-drug interactions and polypharmacy in institutionalized elderly patients in a public hospital in Brazil. *Journal of psychiatric and mental health nursing* 2018;25(1):3-13. doi: 10.1111/jpm.12431 [published Online First: 2017/09/12]
14. Rosholm JU, Bjerrum L, Hallas J, et al. Polypharmacy and the risk of drug-drug interactions among Danish elderly. A prescription database study. *Danish medical bulletin* 1998;45(2):210-3. [published Online First: 1998/05/20]
15. Maher RL, Hanlon J, Hajjar ER. Clinical consequences of polypharmacy in elderly. *Expert Opinion on Drug Safety* 2014;13(1):57-65. doi: 10.1517/14740338.2013.827660
16. Smith SM, Wallace E, O'Dowd T, et al. Interventions for improving outcomes in patients with multimorbidity in primary care and community settings. *The Cochrane database of systematic reviews* 2016;3:Cd006560. doi: 10.1002/14651858.CD006560.pub3 [published Online First: 2016/03/16]
17. Tarn DM, Paterniti DA, Kravitz RL, et al. How Do Physicians Conduct Medication Reviews? *Journal of General Internal Medicine* 2009;24(12):1296. doi: 10.1007/s11606-009-1132-4
18. Mantelli S, Jungo KT, Rozsnyai Z, et al. How general practitioners would deprescribe in frail oldest-old with polypharmacy - the LESS study. *BMC Fam Pract* 2018;19(1):169. doi: 10.1186/s12875-018-0856-9 [published Online First: 2018/10/14]
19. Huiskes VJB, Burger DM, van den Ende CHM, et al. Effectiveness of medication review: a systematic review and meta-analysis of randomized controlled trials. *BMC Family Practice* 2017;18(1):5. doi: 10.1186/s12875-016-0577-x
20. Rankin A, Cadogan CA, Patterson SM, et al. Interventions to improve the appropriate use of polypharmacy for older people. *The Cochrane database of systematic reviews* 2018;9:Cd008165. doi: 10.1002/14651858.CD008165.pub4 [published Online First: 2018/09/04]

For peer review only - http://bmjopen.bmj.com/site/about/guidelines.xhtml

21. Dreischulte T, Donnan P, Grant A, et al. Safer Prescribing--A Trial of Education, Informatics, and Financial Incentives. *The New England journal of medicine* 2016;374(11):1053-64. doi: 10.1056/NEJMsa1508955 [published Online First: 2016/03/18]
22. Scott IA, Pillans PI, Barras M, et al. Using EMR-enabled computerized decision support systems to reduce prescribing of potentially inappropriate medications: a narrative review. *Therapeutic advances in drug safety* 2018;9(9):559-73. doi: 10.1177/2042098618784809
23. Chan AW, Tetzlaff JM, Altman DG, et al. SPIRIT 2013 statement: defining standard protocol items for clinical trials. *Annals of internal medicine* 2013;158(3):200-7. doi: 10.7326/0003-4819-158-3-201302050-00583 [published Online First: 2013/01/09]
24. Huibers MJ, Bleijenberg G, Beurskens AJ, et al. An alternative trial design to overcome validity and recruitment problems in primary care research. *Family practice* 2004;21(2):213-8. doi: 10.1093/fampra/cmh219 [published Online First: 2004/03/17]
25. Yallop JJ, McAvoy BR, Croucher JL, et al. Primary health care research--essential but disadvantaged. *The Medical journal of Australia* 2006;185(2):118-20. [published Online First: 2006/07/18]
26. Jungo K, Löwe A, Mantelli S, et al. Klinische Studie zur Medikamentenoptimierung bei älteren Patient /-innen mit Polypharmazie: Die OPTICA-Studie. *Hospital and Primary Care* 2018;18(8):100–02.
27. Chmiel C, Bhend H, Senn O, et al. The FIRE project: a milestone for research in primary care in Switzerland. *Swiss medical weekly* 2011;140:w13142. doi: 10.4414/smw.2011.13142 [published Online First: 2011/02/01]
28. Drenth-van Maanen AC, Leendertse AJ, Jansen PAF, et al. The Systematic Tool to Reduce Inappropriate Prescribing (STRIP): Combining implicit and explicit prescribing tools to improve appropriate prescribing. *Journal of evaluation in clinical practice* 2018;24(2):317-22. doi: 10.1111/jep.12787 [published Online First: 2017/08/05]
29. Huibers CJA, Sallevelt BTGM, de Groot DA, et al. Conversion of STOPP/START version 2 into coded algorithms for software implementation: A multidisciplinary consensus procedure. *International Journal of Medical Informatics* 2019;125:110-17. doi: doi.org/10.1016/j.ijmedinf.2018.12.010
30. O'Mahony D, O'Sullivan D, Byrne S, et al. STOPP/START criteria for potentially inappropriate prescribing in older people: version 2. *Age and ageing* 2015;44(2):213-18. doi: 10.1093/ageing/afu145
31. Meulendijk MC, Spruit MR, Drenth-van Maanen AC, et al. Computerized Decision Support Improves Medication Review Effectiveness: An Experiment Evaluating the STRIP Assistant's Usability. *Drugs & Aging* 2015;32(6):495-503. doi: 10.1007/s40266-015-0270-0
32. Adam L, Moutzouri E, Loewe A, et al. Rationale and design of OPtimising thERapy to prevent Avoidable hospital admissions in Multimorbid older people (OPERAM): a cluster randomised controlled trial. submitted for publication, 2019.
33. Cowley E, Murphy K, Spruit M, et al. Intervention protocol: Optimising thERapy to prevent Avoidable hospital admission in the Multi-morbid elderly (OPERAM): a structured medication review with support of a computerised decision support system *submitted for publication* 2019
34. Elwyn G, Frosch D, Thomson R, et al. Shared decision making: a model for clinical practice. *J Gen Intern Med* 2012;27(10):1361-7. doi: 10.1007/s11606-012-2077-6 [published Online First: 2012/05/24]
35. Lamers LM. Risk-adjusted capitation payments: developing a diagnostic cost groups classification for the Dutch situation. *Health policy (Amsterdam, Netherlands)* 1998;45(1):15-32. [published Online First: 1998/06/06]
36. Lamers LM, van Vliet RC. The Pharmacy-based Cost Group model: validating and adjusting the classification of medications for chronic conditions to the Dutch situation. *Health policy (Amsterdam, Netherlands)* 2004;68(1):113-21. doi: 10.1016/j.healthpol.2003.09.001 [published Online First: 2004/03/23]
37. Lamers LM, Vliet RC. Health-based risk adjustment Improving the pharmacy-based cost group model to reduce gaming possibilities. *The European journal of health economics : HEPAC : health economics in prevention and care* 2003;4(2):107-14. doi: 10.1007/s10198-002-0159-9 [published Online First: 2004/12/21]
38. von Buedingen F, Hammer MS, Meid AD, et al. Changes in prescribed medicines in older patients with multimorbidity and polypharmacy in general practice. *BMC Family Practice* 2018;19(1):131. doi: 10.1186/s12875-018-0825-3
39. Hanlon JT, Schmader KE, Samsa GP, et al. A method for assessing drug therapy appropriateness. *Journal of clinical epidemiology* 1992;45(10):1045-51. [published Online First: 1992/10/01]
40. Samsa GP, Hanlon JT, Schmader KE, et al. A summated score for the medication appropriateness index: development and assessment of clinimetric properties including content validity. *Journal of clinical epidemiology* 1994;47(8):891-6. [published Online First: 1994/08/01]
41. Dove S, Ruby C, Hanlon J, et al. The effect of hospitalization on suboptimal prescribing in elderly patients. American Society of Consultant Pharmacists 27th Annual Meeting and Exhibition. Nashville, Tenn, 1996.

For peer review only - http://bmjopen.bmj.com/site/about/guidelines.xhtml

42. Jeffery S, Ruby C, Hanlon J, et al. The impact of an interdisciplinary team on suboptimal prescribing in a long term care facility. *Consult Pharm* 1999;14(21):1386–91.
43. Wright RM, Sloane R, Pieper CF, et al. Underuse of indicated medications among physically frail older US veterans at the time of hospital discharge: results of a cross-sectional analysis of data from the Geriatric Evaluation and Management Drug Study. *The American journal of geriatric pharmacotherapy* 2009;7(5):271-80. doi: 10.1016/j.amjopharm.2009.11.002 [published Online First: 2009/12/02]
44. Janssen MF, Pickard AS, Golicki D, et al. Measurement properties of the EQ-5D-5L compared to the EQ-5D-3L across eight patient groups: a multi-country study. *Quality of life research : an international journal of quality of life aspects of treatment, care and rehabilitation* 2013;22(7):1717-27. doi: 10.1007/s11136-012-0322-4 [published Online First: 2012/11/28]
45. EuroQol--a new facility for the measurement of health-related quality of life. *Health policy (Amsterdam, Netherlands)* 1990;16(3):199-208. [published Online First: 1990/11/05]
46. Weinstein MC, Torrance G, McGuire A. QALYs: the basics. *Value in health : the journal of the International Society for Pharmacoeconomics and Outcomes Research* 2009;12 Suppl 1:S5-9. doi: 10.1111/j.1524-4733.2009.00515.x [published Online First: 2009/03/11]
47. Reeve E, Anthony AC, Kouladjian O'Donnell L, et al. Development and pilot testing of the revised Patients' Attitudes Towards Deprescribing questionnaire for people with cognitive impairment. *Australasian journal on ageing* 2018;37(4):E150-e54. doi: 10.1111/ajag.12576 [published Online First: 2018/08/08]
48. Reeve E, Low LF, Shakib S, et al. Development and Validation of the Revised Patients' Attitudes Towards Deprescribing (rPATD) Questionnaire: Versions for Older Adults and Caregivers. *Drugs Aging* 2016;33(12):913-28. doi: 10.1007/s40266-016-0410-1 [published Online First: 2016/10/28]
49. Gallagher PF, O'Connor MN, O'Mahony D. Prevention of potentially inappropriate prescribing for elderly patients: a randomized controlled trial using STOPP/START criteria. *Clinical pharmacology and therapeutics* 2011;89(6):845-54. doi: 10.1038/clpt.2011.44 [published Online First: 2011/04/22]
50. Smeeth L, Ng ES. Intraclass correlation coefficients for cluster randomized trials in primary care: data from the MRC Trial of the Assessment and Management of Older People in the Community. *Controlled clinical trials* 2002;23(4):409-21. [published Online First: 2002/08/06]
51. Eldride S, Kerry S. A Practical Guide to Cluster Randomised Trials in Health Services Research: John Wiley & Sons, Ltd 2012.
52. Buffel du Vaure C, Dechartres A, Battin C, et al. Exclusion of patients with concomitant chronic conditions in ongoing randomised controlled trials targeting 10 common chronic conditions and registered at ClinicalTrials.gov: a systematic review of registration details. *BMJ open* 2016;6(9):e012265. doi: 10.1136/bmjopen-2016-012265
53. World Medical Association Declaration of Helsinki: ethical principles for medical research involving human subjects. *The Journal of the American College of Dentists* 2014;81(3):14-8. [published Online First: 2015/05/09]
54. Guideline for Good Clinical Practice E6(R1): International Council on Harmonisation of Technical Requirements for Registration of Pharmaceuticals for Human Use (ICH), 1996.

For peer review only - http://bmjopen.bmj.com/site/about/guidelines.xhtml

FIGURE LEGENDS

Figure 1: OPTICA trial flow chart

Flow diagram of the progress through the phases of the OPTICA trial. In dark grey: steps done by general practitioners. In white: steps done by study team at BIHAM. Abbreviations: BIHAM, Institute of Primary Health Care of the University of Bern, GPs, general practitioners; STRIPA, Systematic Tool to Reduce Inappropriate Prescribing Assistant / *targeted number

For peer review only

Figure 2: OPTICA data flow chart

Step-by-step explanation of the data flow during the OPTICA trial. Abbreviations: EMR, electronic medical record; FIRE, Family medicine ICPC Research using Electronic medical records; GPs, general practitioners; OPTICA, Optimizing PharmacoTherapy In the Multimorbid Elderly in Primary CAre; STRIPA, Systematic Tool to Reduce Inappropriate Prescribing Assistant

For peer review only

Figure 1: OPTICA trial flow chart

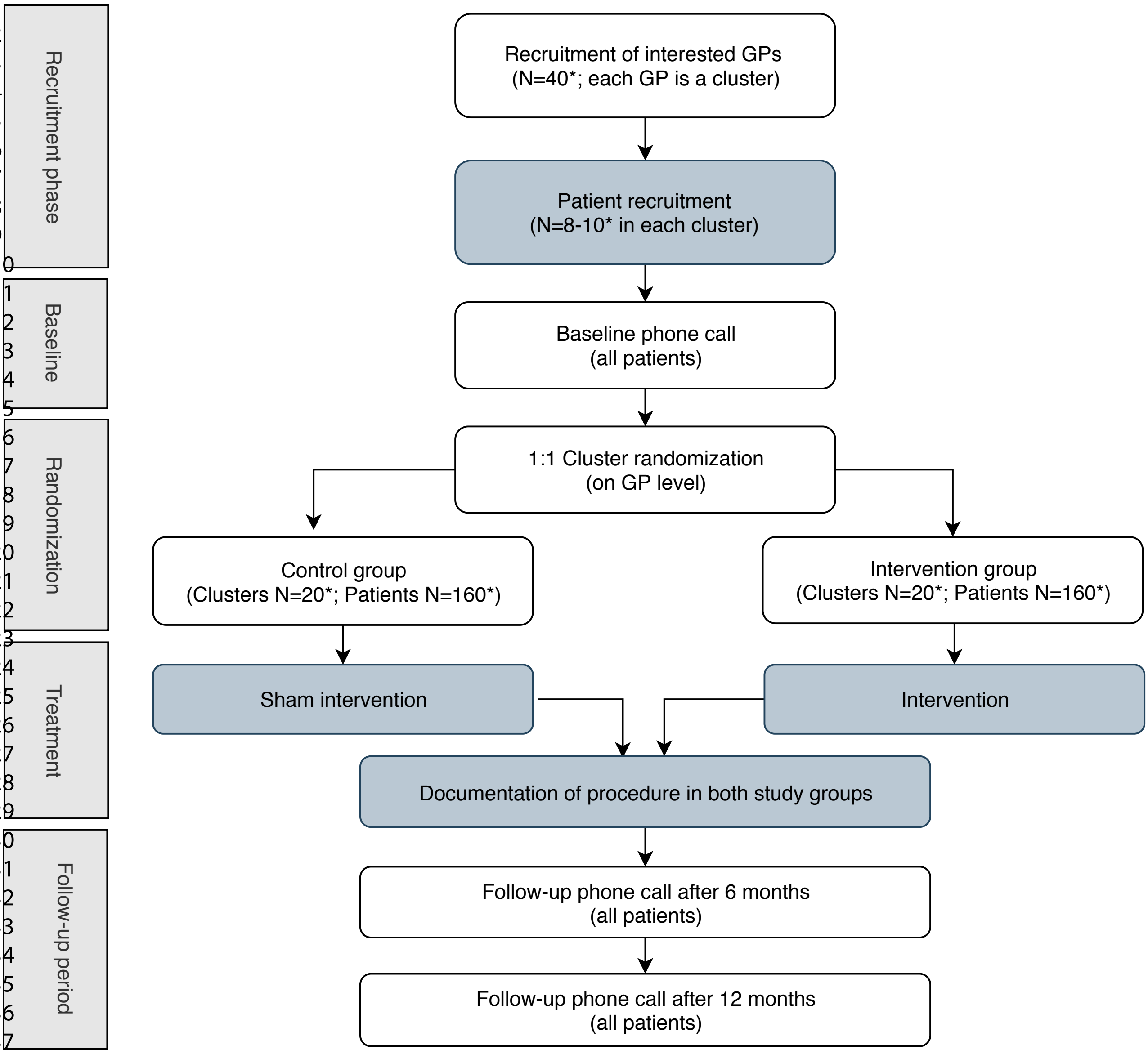

Flow diagram of the progress through the phases of the OPTICA trial. In dark grey: steps done by general practitioners. In white: steps done by study team at BIHAM. Abbreviations: BIHAM, Institute of Primary Health Care of the University of Bern, GPs, general practitioners; STRIPA, Systematic Tool to Reduce Inappropriate Prescribing Assistant / *targeted number

Figure 2: OPTICA data flow chart

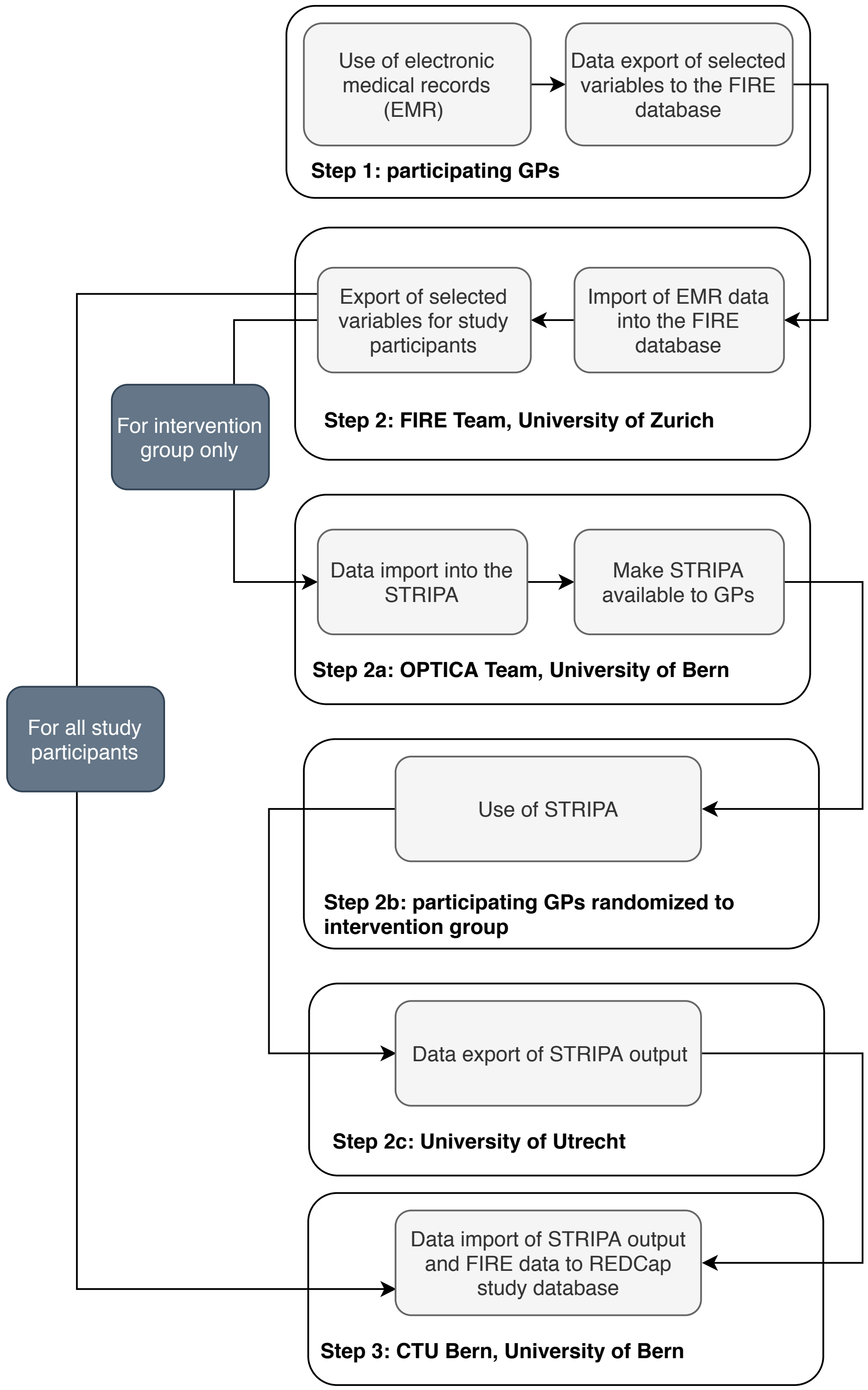

Step-by-step explanation of the data flow during the OPTICA trial. Abbreviations: EMR, electronic medical record; FIRE, Family medicine ICPC Research using Electronic medical records; GPs, general practitioners; OPTICA, Optimizing PharmacoTherapy In the Multimorbid Elderly in Primary CAre; STRIPA, Systematic Tool to Reduce Inappropriate Prescribing Assistant

Reporting checklist for protocol of a clinical trial.

Based on the SPIRIT guidelines.

Instructions to authors

Complete this checklist by entering the page numbers from your manuscript where readers will find each of the items listed below.

Your article may not currently address all the items on the checklist. Please modify your text to include the missing information. If you are certain that an item does not apply, please write "n/a" and provide a short explanation.

Upload your completed checklist as an extra file when you submit to a journal.

In your methods section, say that you used the SPIRIT reporting guidelines, and cite them as:

Chan A-W, Tetzlaff JM, Altman DG, Laupacis A, Gøtzsche PC, Krleža-Jerić K, Hróbjartsson A, Mann H, Dickersin K, Berlin J, Doré C, Parulekar W, Summerskill W, Groves T, Schulz K, Sox H, Rockhold FW, Rennie D, Moher D. SPIRIT 2013 Statement: Defining standard protocol items for clinical trials. Ann Intern Med. 2013;158(3):200-207

		Reporting Item	Page Number
Title	#1	Descriptive title identifying the study design, population, interventions, and, if applicable, trial acronym	p.1
Trial registration	#2a	Trial identifier and registry name. If not yet registered, name of intended registry	p.2
Trial registration: data set	#2b	All items from the World Health Organization Trial Registration Data Set	p.2
Protocol version	#3	Date and version identifier	p.2
Funding	#4	Sources and types of financial, material, and other support	p.2,p.18
Roles and responsibilities: contributorship	#5a	Names, affiliations, and roles of protocol contributors	p.2, p.17-18

For peer review only

Roles and responsibilities: sponsor contact information	#5b	Name and contact information for the trial sponsor	p. 1 (see note 1)
Roles and responsibilities: sponsor and funder	#5c	Role of study sponsor and funders, if any, in study design; collection, management, analysis, and interpretation of data; writing of the report; and the decision to submit the report for publication, including whether they will have ultimate authority over any of these activities	p.17-18 (see note 2)
Roles and responsibilities: committees	#5d	Composition, roles, and responsibilities of the coordinating centre, steering committee, endpoint adjudication committee, data management team, and other individuals or groups overseeing the trial, if applicable (see Item 21a for data monitoring committee)	p. 14, table 9-10
Background and rationale	#6a	Description of research question and justification for undertaking the trial, including summary of relevant studies (published and unpublished) examining benefits and harms for each intervention	p. 4
Background and rationale: choice of comparators	#6b	Explanation for choice of comparators	p.6, p.8
Objectives	#7	Specific objectives or hypotheses	p.4
Trial design	#8	Description of trial design including type of trial (eg, parallel group, crossover, factorial, single group), allocation ratio, and framework (eg, superiority, equivalence, non-inferiority, exploratory)	p.5
Study setting	#9	Description of study settings (eg, community clinic, academic hospital) and list of countries where data will be collected. Reference to where list of study sites can be obtained	p.5
Eligibility criteria	#10	Inclusion and exclusion criteria for participants. If applicable, eligibility criteria for study centres and individuals who will perform the interventions (eg,	p.5-6

For peer review only

		surgeons, psychotherapists)	
Interventions: description	#11a	Interventions for each group with sufficient detail to allow replication, including how and when they will be administered	p.6-8
Interventions: modifications	#11b	Criteria for discontinuing or modifying allocated interventions for a given trial participant (eg, drug dose change in response to harms, participant request, or improving / worsening disease)	p.5-7
Interventions: adherence	#11c	Strategies to improve adherence to intervention protocols, and any procedures for monitoring adherence (eg, drug tablet return; laboratory tests)	p.7-8 (see note 3)
Interventions: concomitant care	#11d	Relevant concomitant care and interventions that are permitted or prohibited during the trial	(see note 4)
Outcomes	#12	Primary, secondary, and other outcomes, including the specific measurement variable (eg, systolic blood pressure), analysis metric (eg, change from baseline, final value, time to event), method of aggregation (eg, median, proportion), and time point for each outcome. Explanation of the clinical relevance of chosen efficacy and harm outcomes is strongly recommended	p.4, p.10-12
Participant timeline	#13	Time schedule of enrolment, interventions (including any run-ins and washouts), assessments, and visits for participants. A schematic diagram is highly recommended (see Figure)	p.5, p.6-8 (see note 5), p. 9
Sample size	#14	Estimated number of participants needed to achieve study objectives and how it was determined, including clinical and statistical assumptions supporting any sample size calculations	p.13
Recruitment	#15	Strategies for achieving adequate participant enrolment to reach target sample size	p.13
Allocation: sequence generation	#16a	Method of generating the allocation sequence (eg, computer-generated random numbers), and list of any factors for stratification. To reduce predictability of a random sequence, details of any planned restriction (eg, blocking) should be provided in a separate	p.5

For peer review only

		document that is unavailable to those who enrol participants or assign interventions	
Allocation concealment mechanism	#16b	Mechanism of implementing the allocation sequence (eg, central telephone; sequentially numbered, opaque, sealed envelopes), describing any steps to conceal the sequence until interventions are assigned	p.5
Allocation: implementation	#16c	Who will generate the allocation sequence, who will enrol participants, and who will assign participants to interventions	p.5
Blinding (masking)	#17a	Who will be blinded after assignment to interventions (eg, trial participants, care providers, outcome assessors, data analysts), and how	p.9-10 (refer to Table 1 in manuscript)
Blinding (masking): emergency unblinding	#17b	If blinded, circumstances under which unblinding is permissible, and procedure for revealing a participant's allocated intervention during the trial	Not applicable
Data collection plan	#18a	Plans for assessment and collection of outcome, baseline, and other trial data, including any related processes to promote data quality (eg, duplicate measurements, training of assessors) and a description of study instruments (eg, questionnaires, laboratory tests) along with their reliability and validity, if known. Reference to where data collection forms can be found, if not in the protocol	p.9, p.10-12
Data collection plan: retention	#18b	Plans to promote participant retention and complete follow-up, including list of any outcome data to be collected for participants who discontinue or deviate from intervention protocols	p.10
Data management	#19	Plans for data entry, coding, security, and storage, including any related processes to promote data quality (eg, double data entry; range checks for data values). Reference to where details of data management procedures can be found, if not in the protocol	p.17
Statistics: outcomes	#20a	Statistical methods for analysing primary and secondary outcomes. Reference to where other	p.13-14

For peer review only

		details of the statistical analysis plan can be found, if not in the protocol	
Statistics: additional analyses	#20b	Methods for any additional analyses (eg, subgroup and adjusted analyses)	Not applicable.
Statistics: analysis population and missing data	#20c	Definition of analysis population relating to protocol non-adherence (eg, as randomised analysis), and any statistical methods to handle missing data (eg, multiple imputation)	p.13
Data monitoring: formal committee	#21a	Composition of data monitoring committee (DMC); summary of its role and reporting structure; statement of whether it is independent from the sponsor and competing interests; and reference to where further details about its charter can be found, if not in the protocol. Alternatively, an explanation of why a DMC is not needed	p.14
Data monitoring: interim analysis	#21b	Description of any interim analyses and stopping guidelines, including who will have access to these interim results and make the final decision to terminate the trial	p.6 (See note 6)
Harms	#22	Plans for collecting, assessing, reporting, and managing solicited and spontaneously reported adverse events and other unintended effects of trial interventions or trial conduct	p.12
Auditing	#23	Frequency and procedures for auditing trial conduct, if any, and whether the process will be independent from investigators and the sponsor	p.12
Research ethics approval	#24	Plans for seeking research ethics committee / institutional review board (REC / IRB) approval	p. 17, p.18 (see note 8)
Protocol amendments	#25	Plans for communicating important protocol modifications (eg, changes to eligibility criteria, outcomes, analyses) to relevant parties (eg, investigators, REC / IRBs, trial participants, trial registries, journals, regulators)	p.17
Consent or assent	#26a	Who will obtain informed consent or assent from potential trial participants or authorised surrogates,	p.6, p.9

For peer review only

		and how (see Item 32)	
Consent or assent: ancillary studies	#26b	Additional consent provisions for collection and use of participant data and biological specimens in ancillary studies, if applicable	p.6
Confidentiality	#27	How personal information about potential and enrolled participants will be collected, shared, and maintained in order to protect confidentiality before, during, and after the trial	p.17
Declaration of interests	#28	Financial and other competing interests for principal investigators for the overall trial and each study site	p.18
Data access	#29	Statement of who will have access to the final trial dataset, and disclosure of contractual agreements that limit such access for investigators	p.17
Ancillary and post trial care	#30	Provisions, if any, for ancillary and post-trial care, and for compensation to those who suffer harm from trial participation	p.17
Dissemination policy: trial results	#31a	Plans for investigators and sponsor to communicate trial results to participants, healthcare professionals, the public, and other relevant groups (eg, via publication, reporting in results databases, or other data sharing arrangements), including any publication restrictions	p.17
Dissemination policy: authorship	#31b	Authorship eligibility guidelines and any intended use of professional writers	p.17
Dissemination policy: reproducible research	#31c	Plans, if any, for granting public access to the full protocol, participant-level dataset, and statistical code	p.17, p. 18
Informed consent materials	#32	Model consent form and other related documentation given to participants and authorised surrogates	see note 7
Biological specimens	#33	Plans for collection, laboratory evaluation, and storage of biological specimens for genetic or molecular analysis in the current trial and for future use in ancillary studies, if applicable	Not applicable.

For peer review only

Author notes

1. Prof. Sven Streit is the Sponsor-Investigator of the OPTICA trial
2. The role of the Sponsor Investigator, Prof. Sven Streit, is described on p. 17. The funder, the Swiss National Science Foundation, does not have any role in “collection, management, analysis, and interpretation of data; writing of the report; and the decision to submit the report for publication”.
3. GPs record in the REDCap study database if and how they have performed the intervention.
4. Usual care is permitted.
5. Refer to Figure 1.
6. There will be no interim analyses.
7. Available upon request. All documents are in German.
8. Approval received in autumn 2018.

The SPIRIT checklist is distributed under the terms of the Creative Commons Attribution License CC-BY-ND 3.0. This checklist was completed on 02. April 2019 using https://www.goodreports.org/, a tool made by the EQUATOR Network in collaboration with Penelope.ai

For peer review only
